# Multifaceted roles of Galectins: from carbohydrate binding to targeted cancer therapy

**DOI:** 10.1186/s40364-025-00759-1

**Published:** 2025-03-25

**Authors:** Nan Zhang, Qiao Liu, Daihan Wang, Xiaoyun Wang, Zhaoping Pan, Bo Han, Gu He

**Affiliations:** 1 State Key Laboratory of Southwestern Chinese Medicine Resources, College of Medical Technology and School of Pharmacy, Chengdu University of Traditional Chinese Medicine, Chengdu, 611137 China; 2 Institute of Precision Drug Innovation and Cancer Center, the Second Hospital of Dalian Medical University, Dalian, 116023 China

**Keywords:** Galectin, Glycan, Tumor microenvironment, Immune tolerance, Targeted therapy

## Abstract

Galectins play pivotal roles in cellular recognition and signaling processes by interacting with glycoconjugates. Extensive research has highlighted the significance of Galectins in the context of cancer, aiding in the identification of biomarkers for early detection, personalized therapy, and predicting treatment responses. This review offers a comprehensive overview of the structural characteristics, ligand-binding properties, and interacting proteins of Galectins. We delve into their biological functions and examine their roles across various cancer types. Galectins, characterized by a conserved carbohydrate recognition domain (CRD), are divided into prototype, tandem-repeat, and chimera types based on their structural configurations. Prototype Galectins contain a single CRD, tandem-repeat Galectins contain two distinct CRDs linked by a peptide, and the chimera-type Galectin-3 features a unique structural arrangement. The capacity of Galectins to engage in multivalent interactions allows them to regulate a variety of signaling pathways, thereby affecting cell fate and function. In cancer, Galectins contribute to tumor cell transformation, angiogenesis, immune evasion, and metastasis, making them critical targets for therapeutic intervention. This review discusses the multifaceted roles of Galectins in cancer progression and explores current advancements in the development of Galectin-targeted therapies. We also address the challenges and future directions for integrating Galectin research into clinical practice to enhance cancer treatment outcomes. In brief, understanding the complex functions of Galectins in cancer biology opens new avenues for therapeutic strategies. Continued research on Galectin interactions and their pathological roles is essential for developing effective carbohydrate-based treatments and improving clinical interventions for cancer patients.

**Graphical Abstract:**

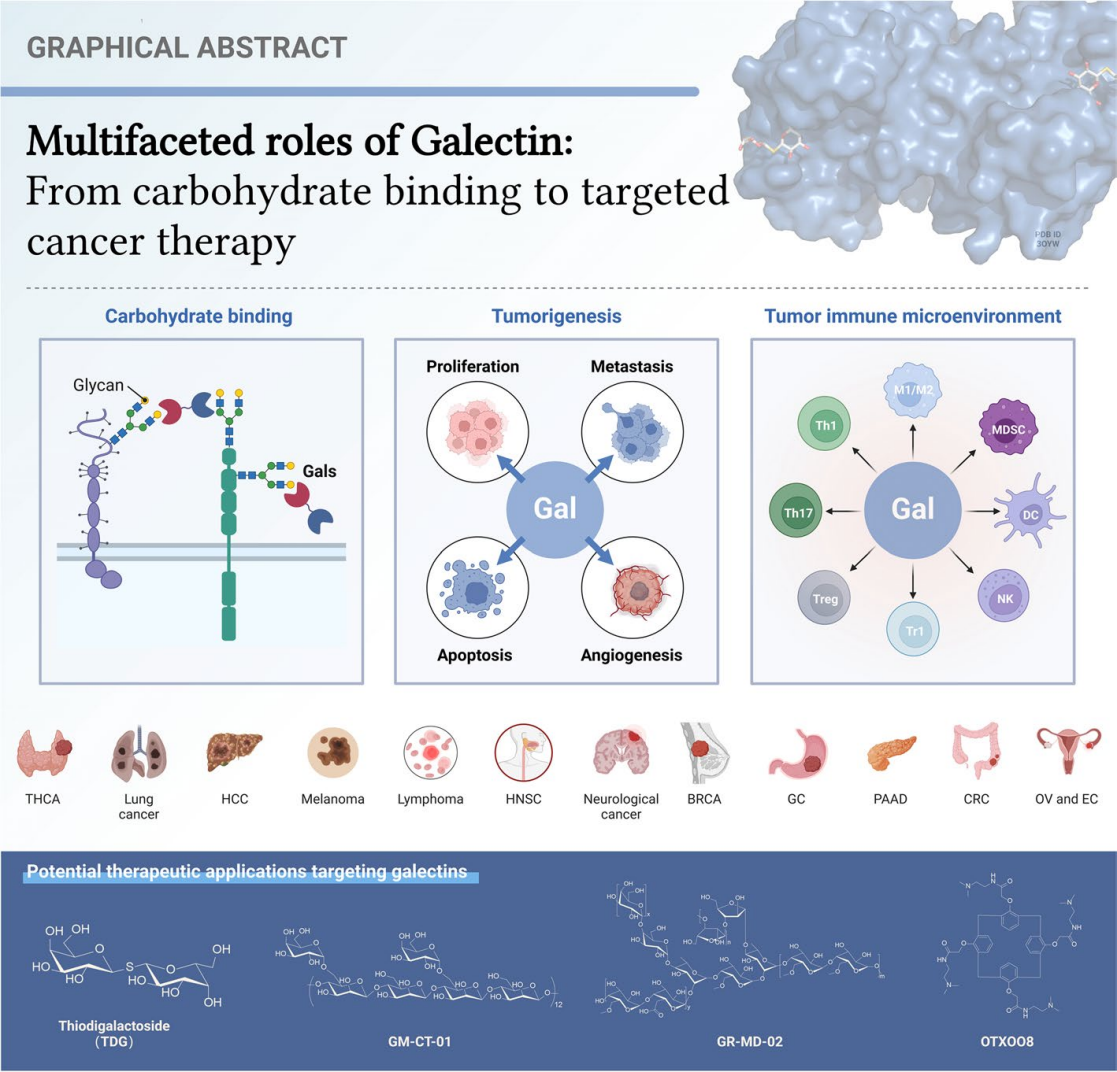

## Background

Mammals express various glycan-binding proteins (GBPs) that recognize glycoconjugates with diverse surface modifications on cells, thereby regulating multiple receptors and mediating complex signal transduction both inside and outside the cell. Extensive research on the cancer-specific and organ-specific changes of polysaccharides and glycoproteins in glycobiology facilitates the identification and creation of biomarkers for detecting cancer at an early stage. These biomarkers play a crucial role in early cancer intervention, personalized treatment decision-making, and predicting treatment outcomes, including responses to immunotherapy.

In humans, over eighty glycan-binding proteins have been identified and categorized into various structural families, each characterized by a conserved Carbohydrate Recognition Domain (CRD). Galectins, part of the β-galactoside-binding protein family, are secreted directly from the cytoplasm into the extracellular environment, by passing the classical endoplasmic reticulum/Golgi transport mechanisms. Galectins primarily operate by interacting with specific glycoconjugates, which consist of carbohydrate structures attached to proteins, peptides, and lipids. These interactions allow galectins to interpret and convert the information encoded by glycosylation into cellular activities. So far, sixteen distinct galectin members have been discovered in mammals, with twelve identified in humans, including Galectin-1, −2, −3, −4, −7, −8, −9, −10, −12, −13, −14, and −16.

**Fig. 1:**
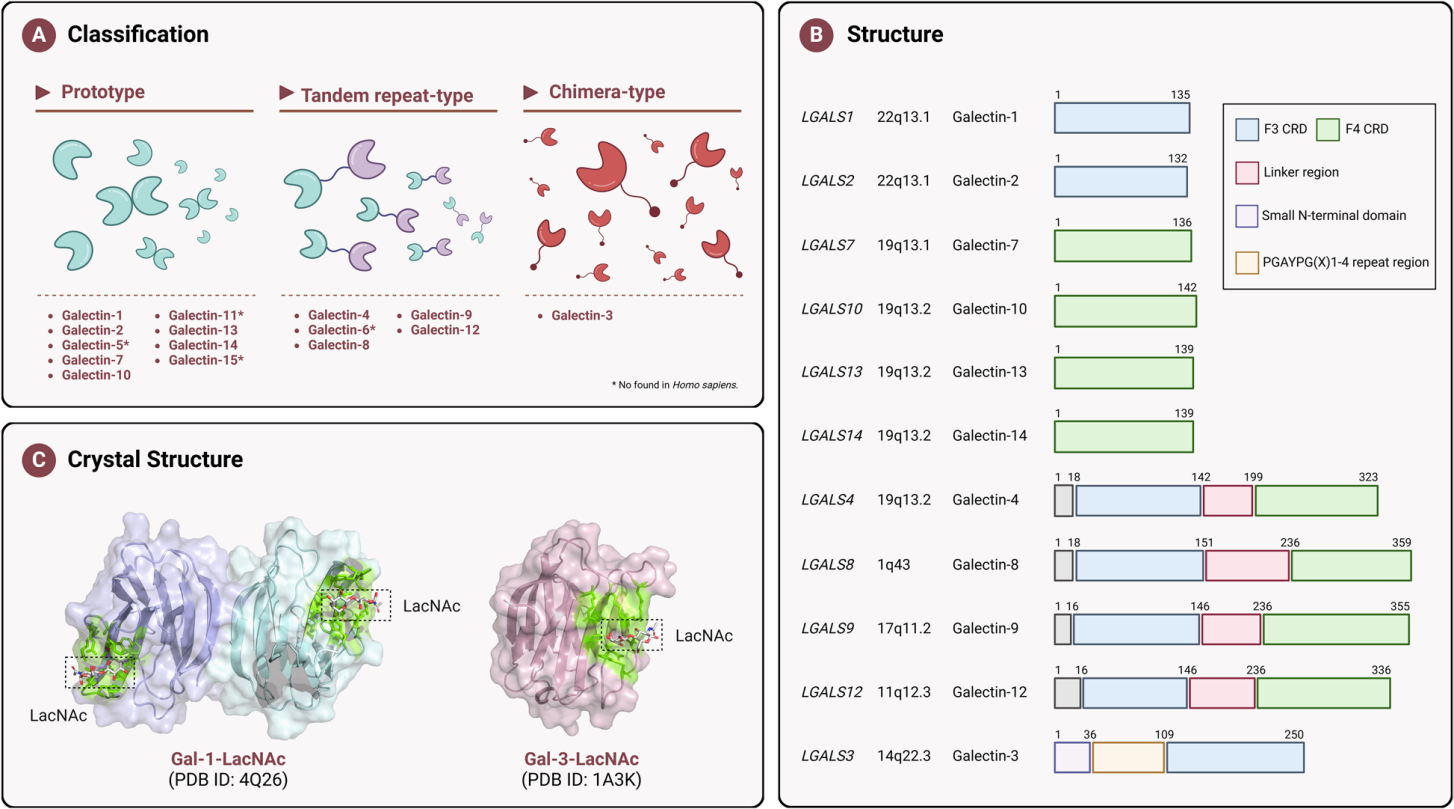
Diverse structures of galectins. **a** Galectins can be subdivided into three subgroups: prototype galectins (Gal-1, −2, −5, −7, −10, −11, −13, −14, −15) are homodimers, consisting of two identical CRDs bound together by electrostatic forces. Tandem repeattype galectins (Gal-4, −6, −8, −9, −12) possess two different CRDs (N-terminal and C-terminal) joined by a linker polypeptide. The unique chimera-type Gal-3 composed of unusual tandem repeats of proline- and glycine-rich short stretches fused onto the CRD. Multivalent ligands can promote conformational changes and oligomerization to form pentamers. **b** Schematic representation of protein domains of different members of the Galectins family. **c** Crystals of of human Gal-1 (left, PDB-ID: 4Q26, resolution: 1.40 Å) and Gal-3 (right, PDB-ID: 1A3K, resolution: 2.10 Å) bound to N-acetyllactosamine (Galb4GlcNAc, LacNAc; carbons in grey, oxygens in red, and nitrogens in blue). Amino acids that directly interact with bound carbohydrates are highly conserved and are primarily distributed on the β-sheet. In Gal-1, the key amino acid residues involved in LacNAc binding are His44, Asn46, Arg48, His52, Asp54, Val59, Asn61, Trp68, Glu71, and Arg73. In Gal-3, the corresponding residues are Arg144, His158, Asn160, Arg162, Glu165, Asn174, Trp181, Glu184, and Arg186. The surface involved in these interactions is depicted in green. CRD: Carbohydrate recognition domains

Galectins have a similar structural fold, featuring an approximately 130-amino-acid conserved CRD essential for carbohydrate binding. They are traditionally classified based on protein structure similarities into three types: prototype galectins, tandem-repeat galectins, and chimera-type galectins. Prototype galectins, such as Galectin-1, −2, −5, −7, −10, −11, −13, −14, and −15, have one CRD and form monomers or non-covalent homodimers. Tandem-repeat galectins, like Galectin-4, −6, −8, −9, and −12, are heterodimers with two distinct CRDs linked by a peptide of up to 70 amino acids. Chimera-type Galectin-3 has a CRD at the end of a chain 120–160 amino acids long and forms a pentamer (Fig. 1). Most galectins are divalent or multivalent, enabling them to interact with multiple binding partners and trigger various signaling pathways. Prototype galectins dimerize, tandem-repeat galectins are at least divalent, and Galectin-3 oligomerizes when binding multivalent glycoproteins.

**Fig. 2:**
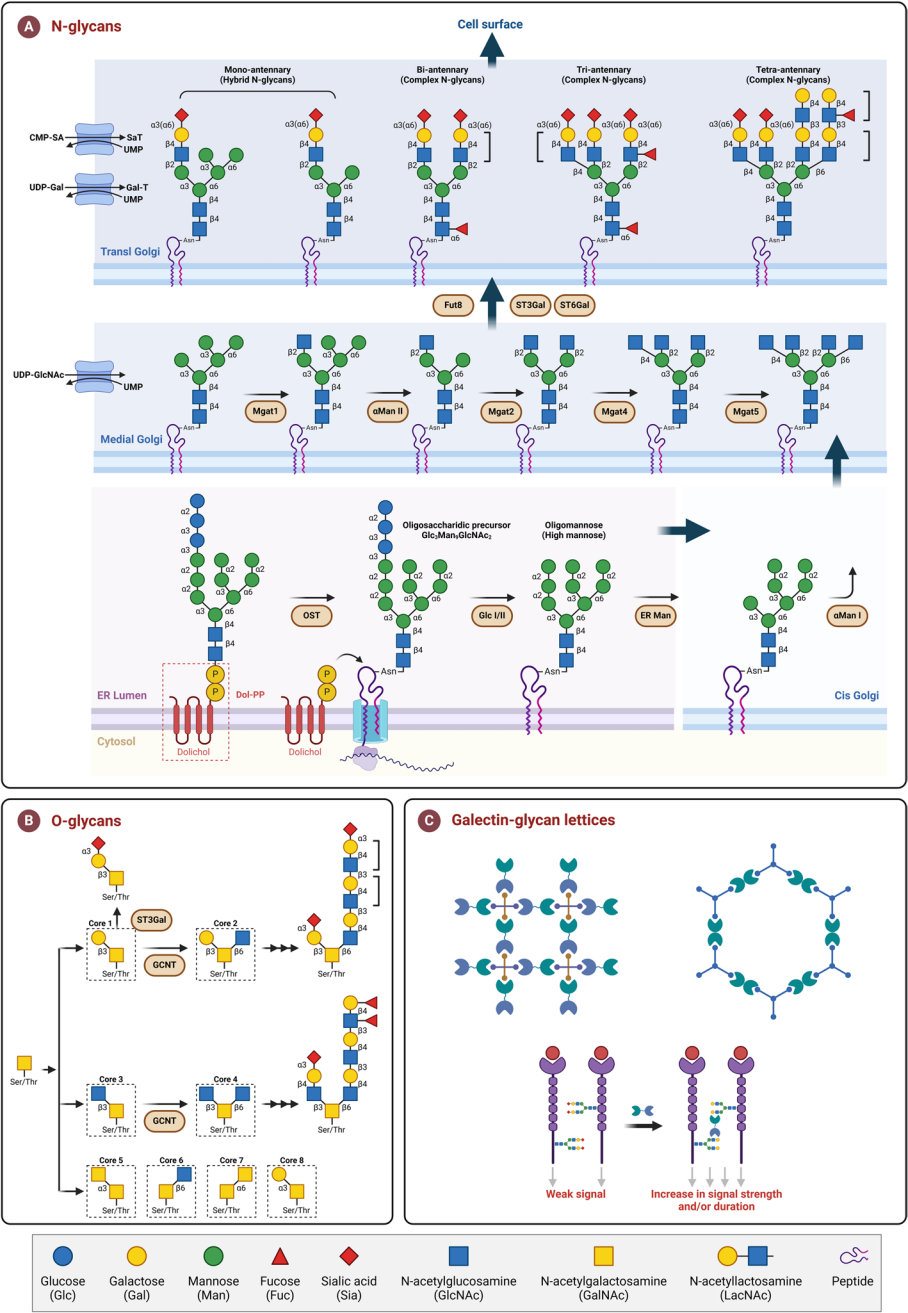
N- and O-glycan biosynthesis, and Galectin Lattice. **a** N-glycan formation begins with the modification of the conserved core structure Glc3Man9GlcNAc2 with polysaccharide "antennae," resulting in three types of structures: hybrid, high mannose, and complex. OST transfers the pre-assembled donor Glc3Man9GlcNAc2 from the phosphatidylglycerol lipid anchor (dolichol PP) to the Asn residue of the consensus sequence NXS/T (where X ≠ P) motif of nascent glycoproteins in the ER. These glycoproteins are then modified by glucosidase I, glucosidase II, and ER α-mannosidase, and further mature as they progress along the secretory pathway. Properly folded glycoproteins are transported from the ER to the cis, medial, and trans Golgi apparatus, where they undergo additional modifications by α-mannosidase I, α-mannosidase II, and N-acetylglucosamine transferase (Mgat), completing the branching and extension processes in the Golgi. The glycoproteins are further decorated with sialic acid and fucose residues, resulting in a diverse and structurally complex array of glycosylation modifications. Finally, mature glycoproteins are transported to the cell surface. The terminal sialic acid residues reduce the affinity of N-glycan for galectins, whereas the addition of GalNAc increases it. Individual galectins exhibit refined specificity for particular polysaccharide structures, as reviewed in detail in the literature. **b** Under normal circumstances, N-glycans are first produced in the endoplasmic reticulum, whereas O-glycans are produced in the Golgi apparatus. O-GalNAc represents a large class of O-linked extracellular polysaccharides. These structures are formed by a GalNAc residue linked to a Ser/Thr residue, creating eight different core structures through the action of various glycosyltransferases, such as core 2 N-acetylglucosamine transferase 1 (GCNT1) and α 2,3 sialyltransferase 1 (ST3Gal1). **c** Different types of crystal lattices can form between multivalent galectins and multivalent polysaccharides. A single receptor binds to a single ligand with low affinity, resulting in a weak signal for each combination. However, appropriate glycosylation modifications can enable receptor cross-linking mediated by galectins. This multivalent affinity between galectins and sugar ligands can significantly enhance the overall affinity, thereby increasing the intensity and/or duration of the signal. The monosaccharides in polysaccharides are represented by specific symbols in the Symbol Nomenclature for Glycans (SNFG) through illustrative images. OST: Oligosaccharyltransferase; ER: Endoplasmic reticulum; Glc I/II: Glucosidase I, II; ER Man: ER α-mannosidase; Mgat1: N-acetylglucosaminyltransferase I (GnT-I); αMan II: α-mannosidase II (MAN2A1 and MAN2A2); Fut8: α1,6-fucosyltransferase

Galectins exist in many tissues and significantly impact cell fate. As soluble endogenous lectins, they are believed to engage with cell surface glycans outside cells, modulating signal transduction and influencing cell destiny (Fig. 2). During cancer progression, galectins act like cytokines and growth factors, playing crucial roles. They aid in tumor cell transformation, enhance angiogenesis, suppress immune responses against tumors, and assist in metastasis. Consequently, they are regarded as versatile targets in cancer treatment. Studying the functions of galectins can reveal new pathological features and mechanisms of disease, offering new avenues for carbohydrate-based disease treatment. This review highlights major progress in research on the galectin family and examines the potential and challenges of their clinical application to enhance cancer therapy.

## Galectins: structural and subcellular localization perspectives

### Structure and biochemical characteristics

Galectins have a common affinity for β-galactoside sugars and show significant sequence similarity within their CRDs. Each member of this family features a conserved CRD with a β-galactoside affinity. The CRD, the main functional domain, consists of about 130 amino acids, forming two β-sheets that create a β-sandwich pocket. Five antiparallel strands (F1-F5) form the convex face, while six antiparallel strands (S1-S6) make up the concave face. Carbohydrate binding takes place on the concave side within the groove shaped by the sandwich's curvature. The core site for β-galactoside-containing disaccharides like lactose is comprised of conserved amino acids in the S4–S6 strands; however, the binding groove extends to strands S1–S3, accommodating larger oligosaccharides with varied affinities. Prior to or early in chordate evolution, duplication of the single CRD galectin gene led to the ancestral double CRD gene. The N-terminal and C-terminal CRDs subsequently evolved into two distinct subtypes, defined by exon–intron structures (F4-CRD and F3-CRD). Single CRD galectins are categorized into F4-CRD galectins—including LGALS-7, LGALS-10, LGALS-11, LGALS-13, LGALS-14, LGALS-15—and F3-CRD galectins, such as LGALS-1, LGALS-2, LGALS-3, and LGALS-5 (Fig. 1B). Notably, among the other five double CRD galectins, the N-terminal CRD is the F4 subtype, while the C-terminal CRD is the F3 subtype [1, 2].

#### Galectin-1

The gene encoding Galectin-1 (Gal-1), *LGALS1*, is located on chromosome 22q13.1 (Fig. 1B) [3]. This gene is composed of four exons that are spliced to form a 0.6 kb transcript (GenBank: NM_002305). The resulting translation product is the Gal-1 protein with a molecular weight of 14.5 kDa. Gal-1 is a prototype galectin, composed of a single CRD subunit. The CRD structure in Gal-1 protein is highly conserved across many vertebrates. Gal-1 can be found both as a monomer and a non-covalent homodimer. The monomer consists of a β-sandwich structure made up of five F-strands (F1-F5) and six S-strands (S1-S6a/b) forming an antiparallel β-sheet. This structure creates the carbohydrate-binding groove. The N- and C-termini of each monomer are positioned at the dimer interface, while the glycan-binding sites are located at opposite ends of the dimer. The integrity of the dimeric form is primarily maintained through hydrophobic interactions within the hydrophobic core at the monomer–monomer interface [4]. This hydrophobic core is established by the side chains of Leu4, Ala6, Ile128, Val131, and Phe133 from two subunits, creating a solid structure [5]. The main chains of residues Val5, Ser7, Val131, Lys129, and Phe133 from both subunits create a distinct hydrogen bond network [5]. The Gal-1 sequence contains six cysteine residues that can be either reduced or oxidized. When oxidized, these cysteines form three intramolecular disulfide bonds (Cys2-Cys130, Cys16-Cys88, Cys42-Cys60), resulting in conformational changes that diminish lectin activity (CRD capability) [6]. In its oxidized state, Galectin-1 becomes a monomer with no lectin activity but promotes axonal regeneration [7]. Conversely, reduced Galectin-1 forms a dimer with strong CRD binding capacity, playing roles in cell adhesion, migration, proliferation, survival, immunosuppression, and angiogenesis [8].

#### Galectin-2

Galectin-2 (Gal-2), like Gal-1, is a prototype galectin. Discovered in 1992 within the cDNA library of human liver cancer cell HepG2, *LGALS2* is positioned on the opposite strand of the same chromosome as LGALS1, approximately 50 kb away (Fig. 1B). Gal-2, a 14 kDa protein, features a CRD at its C-terminus [9]. It displays a β-sandwich structure formed by two β-sheets, with one containing five β-strands (F1-F5) and the other six (S1-S6), surrounding the CRD on either side. [10] (Fig. 1). Gal-2 can exist both as a monomer and as non-covalent homodimers, allowing it to cross-link receptors on the cell surface [10]. Its amino acid sequence shares 43% homology with Gal-1, indicating structural similarity, although Gal-2 expression is predominantly localized to the gastrointestinal tract, with detections also noted in placental and cardiovascular tissues among others.

#### Galectin-3

The protein Galectin-3 (Gal-3) is encoded by the *LGALS3* gene, which is situated on chromosome 14 at positions q21-q22 (Fig. 1B). The promoter region of this gene includes several regulatory elements, such as Sp1 binding sites, the AP-1 complex, a cAMP-responsive element (CRE), and two NF-kB-like sites [11]. Gal-3, a distinct member of the galectin family, is the only chimeric-type galectin with a molecular weight of 35 kDa. It features a brief N-terminal domain (ND) and a globular C-terminal domain (CD) containing the carbohydrate recognition motif and an NWGR anti-apoptotic motif [12].

The ND of Gal-3 is vital for its oligomerization, secretion, and nuclear translocation. It includes a 12-amino acid leader sequence featuring casein kinase I serine phosphorylation sites, a conserved sequence unique to the galectin family [13]. In some reports, Gal-3 has been shown to precipitate from solution as a pentamer when interacting with certain synthetic multivalent carbohydrates via interactions between its non-lectin N-terminal domains. However, the existence of Gal-3 in pentameric form lacks experimental validation. In studies by Prof. Tai’s group, it was demonstrated using liquid–liquid phase separation (LLPS) that Gal-3 indeed forms oligomers by interacting with various cell surface proteins. This is primarily mediated by dynamic interactions between Gal-3’s proline-rich N-terminal tail (NT) and its CRD. These interactions facilitate the dynamic regulation of Gal-3 on the function of glycosylated cell surface receptors [14–16].

The CD contains a CRD that specifically interacts with multiple N-acetyllactosamine residues on laminin, facilitating interactions with glycoconjugates containing N-acetylglucosamine. Interestingly, despite Gal-3 not belonging to the Bcl-2 family, its CRD contains a four-amino acid sequence, Asn-Trp-Gly-Arg (NWGR) (residues 180–183). This sequence, which plays an anti-apoptotic role in cells, is highly conserved in the BH-1 domain of the Bcl-2 family and is absent in other members of the galectin family. The NWGR motif in Gal-3 interacts with Bcl-2, regulating the release of cytochrome c and cell cycle processes, and participating in anti-apoptotic processes. Gal-3 cross-links with glycosylated ligands, creating dynamic cellular surface lattices that regulate the positioning and endocytosis of glycoproteins and glycolipids in the plasma membrane [17].

Gal-3 is both an intracellular and extracellular lectin, predominantly located in the cytoplasm with a significant distribution in the nucleus as well. It is secreted through a non-classical secretion pathway, and exosomes may be involved in its secretion process [18, 19]. The secretion of Gal-3 relies on oligomerization. The deletion of the N-terminal (the first 12 amino acid residues) prevents Gal-3 from being secreted into the cell supernatant. This mechanism is unique to Gal-3, although all members of the galectin family are secreted through non-conventional pathways [18].Gal-3 binds to cell surface matrix glycoproteins bearing galactose to facilitate cell migration and shuttles between the nucleus and cytoplasm, thereby protecting specific cells from stress. The serine phosphorylation sites in the ND are critical for nuclear transport, while the nuclear localization sequence (NLS) and nuclear export sequence (NES) in the CD determine its cellular positioning. Gal-3 is transported to the nucleus by importin-α/β and is recognized by the CRM1 export protein for its nuclear-cytoplasmic movement [13].

#### Galectin-4

Galectin-4 (Gal-4), initially discovered as a 32 kDa galectin from the *C. elegans*, represents the first subfamily of galectins characterized by two CRDs within a single peptide chain, belonging to the tandem-repeat type of galectins [20]. Human Gal-4, encoded by the *LGALS4* gene on chromosome 19 at q13.1–2 (NCBI Gene ID: 3960; https://www.ncbi.nlm.nih.gov/gene/3960), comprises 10 exons and encodes a protein with 323 amino acids (Fig. 1B). This protein features two carbohydrate recognition domains: the ND (Gal-4N) and the C-terminal carbohydrate-binding domain (Gal-4C), connected by a peptide bridge known as the linking region. These two CRDs exhibit distinct carbohydrate-binding specificities and are expected to show preferences for different ligand groups. Gal-4 is uniquely expressed in the digestive tract, ranging from the tongue to the colon, in both normal developmental and adult tissues [21].

#### Galectin-7

Galectin-7 (Gal-7), a prototype of the galectin family, is encoded by the *LGALS7* gene on chromosome 19q13.2 and has a molecular weight of 15 kDa (Fig. 1B). Similar to other galectins, Gal-7 lacks a signal peptide necessary for secretion, and therefore, is secreted via a non-classical pathway [22]. Gal-7 can form homodimers with distinct topological structures. In contrast to prototype galectins like Gal-1 or Gal-2, which bind in a "side-by-side" manner, Gal-7 generally forms homodimers in a "back-to-back" configuration, providing a larger dimer interface than other prototype galectin homodimers. Like other galectins, Gal-7 can bind to a variety of potential receptors, including internal N-acetyllactosamine (LacNac) oligosaccharide residues and non-reducing end LacNac residues [23].

Gal-7 shows highly tissue-specific expression, mainly present in stratified epithelial cells of the tongue, esophagus, lips, and epidermis, as well as the oral epithelium, cornea, Hassall's corpuscles in the thymus, the urogenital system, the stratified squamous epithelium of the stomach, and the myoepithelial cells of the mammary gland [24]. Typically, Gal-7 subcellular localization is concentrated in the cytoplasm, nucleus, and mitochondria. A mutation at residue 74 amino acids can induce Gal-7 relocalization to mitochondria and nuclear compartments, reducing its affinity for glycoprotein binding.

#### Galectin-8

Galectin-8 (Gal-8), initially cloned from a rat liver cDNA library, is among the most commonly expressed galectins in both normal and cancerous human tissues. It is encoded by *LGALS8* located on human chromosome 1, forming a protein of approximately 34 kDa (Fig. 1B) [25]. *LGALS8* encodes at least four isoforms, with varying peptide lengths (24–74 amino acids) connecting the two CRDs of different isoforms. Variations in the spacing between the two CRDs might affect the carbohydrate binding specificity, resulting in distinct functional roles [26]. Gal-8, which also lacks an N-terminal signal sequence, is secreted through non-classical pathways [26]. Gal-8 is found in bodily fluids such as synovial fluid of rheumatoid arthritis patients, as well as in breast serum and colon serum of cancer patients [26].

#### Galectin-9

Galectin-9 (Gal-9), encoded by the *LGALS9* gene on the short arm of chromosome 17 (17q11.2), consists of 11 exons and produces a protein with a molecular weight of 36 kDa (Fig. 1B). Alternative splicing of *LGALS9* transcripts results in three classic isoforms with variable lengths of flexible linker peptides. Initially isolated from mouse embryonic kidney tissue in 1997, Gal-9 was later cloned from tumor tissues of nodular sclerosing Hodgkin's lymphoma [27]. Gal-9, characterized as a tandem-repeat type galectin, comprises two homologous CRDs: N-CRD and C-CRD. The N-CRD has 148 amino acids and shares 39% sequence identity with the 149-amino acid C-CRD. X-ray crystallography shows that the N-CRD includes a short α-helix within a β-sandwich motif, flanked by six (S1-S6) and five (F1-F5) β-strands. The C-CRD similarly features two antiparallel S1-S6 and F1-F5 β-strands, accompanied by an α-helix [28]. Both N- and C-CRDs feature carbohydrate-binding pockets specific to β-galactosides, with distinct amino acid sequences in the S4, S5, and S6 β-strands contributing to different affinities and physiological activities. The N-CRD primarily activates dendritic cells, while the C-CRD governs receptor recognition and signal transduction in T cell death pathways. The flexible linker peptide between the two CRDs allows for rotation, facilitating diverse polysaccharide-Gal-9 complex formation. Protease-sensitive sites within the linker peptide enable Galectin-9 to be cleaved into monovalent sugar-specific proteins akin to galectin prototypes [29]. Recent studies suggest that Galectin-9 is extensively present in numerous tissues like the liver, spleen, stomach, colon, lymph nodes, appendix, gallbladder, bone marrow, lung, and bladder. It is also found in various cell types, including eosinophils, epithelial cells, endothelial cells, T lymphocytes, dendritic cells, and macrophages.

#### Galectin-10

Galectin-10 (Gal-10) is located on human chromosome 19q13.2 (http://www.genecards.org). The transcription start site of the *LGALS10* gene is located 43 bp upstream of the cDNA sequence's 5′ end (Fig. 1B). This upstream region contains various transcription factor binding sites, including GATA-1, PU.1, Oct, Sp.1, EoTF, NF-1, AP-2, and AML1, which regulate the expression of Gal-10 [30].

Gal-10, a member of the prototype galectins, has its CRD encoded by a single exon. Gel filtration chromatography shows that Gal-10 can dimerize, but its dimeric structure is distinct from other prototype galectins. An α-helix links the S3 and F2 β-strands, directly interacting with the opposite subunit's S1, S2, and S3 β-strands. The two CRDs of Gal-10 form a dimer with their S faces facing each other, unlike other prototype galectin dimers where the S face is exposed for sugar binding. The S-to-S face dimer configuration of Gal-10 may influence its specificity for binding ligands [31]. Biochemical analyses using solid-phase monosaccharides reveal that Gal-10 exhibits weak specific binding to N-acetyl-D-glucosamine and lactose, potentially due to interactions with agarose substrates. Crystal structure studies suggest that Gal-10 does not bind β-galactosides but can interact with mannose. Additionally, some reports indicate that Gal-10 can bind flexible glycerol. Overall, studies on the natural carbohydrate ligands of Gal-10 have not yet reached a definitive conclusion.

Gal-10 mRNA is abundantly transcribed in bone marrow, indicating that Gal-10 might be crucial for lymphocyte maturation. Gal-10 is one of the most abundant cytoplasmic proteins in human eosinophils and can be observed forming Charcot–Leyden crystals in various eosinophilic cell diseases. Gal-10 is found in the nucleus, cytoplasm, granules, and extracellular matrix (ECM), and it can attach to glycans on T-cell membranes, inhibiting the activity of human CD4 + CD25 + regulatory T cells. Galectin-10 is not released into the extracellular space via the classic secretion pathway but rather through extracellular trap cell death (ETosis), where the rapid decomposition of the eosinophil cell membrane during ETosis releases a large amount of Gal-10, leading to increased levels of Gal-10 in the serum and tissues of eosinophils [32]. Galectins may also be present in the extracellular space, with Galectin-3, −6, and −9 identified in extracellular vesicles (EVs). Additionally, mass spectrometry (MS) analysis has revealed the internalization of Gal-10 within EVs [33, 34]. While some clear studies on the intracellular distribution and release mechanisms of Gal-10 exist, many questions remain unresolved about the specific ligands it binds in vivo and how it functions in signal transduction [32].

#### Galectin-5、6、11、15

Galectin-5 (Gal-5) is an N-terminal prototype galectin found exclusively in rats and exists as a monomer in solution. Initially purified from rat lung tissue and termed RL-18, Gal-5 was discovered through sequencing of the rat reticulocyte cDNA library, revealing over 80% homology between rat Gal-5 and the C-CRD of rat Gal-9. Gal-5 lacks signal peptide and shares similar protein sizes with Gal-1 and Gal-2. Unlike Gal-1 and Gal-2, Gal-5 under non-denaturing conditions behaves as a monomer on gel filtration but has the ability to form oligomers. Cell surface glycans serve as functional docking sites for lectins within the galectin family, mediating a variety of responses through interactions with polysaccharides. Studies examining Gal-5's binding affinity to natural polysaccharides reveal strong interactions with glycoproteins (GPs), particularly preferring Galβ1–3/4GlcNAc termini, predominantly found on erythrocytes [35].

Research on Galectin-6 (Gal-6) remains limited, identified as a novel galectin primarily expressed in the mouse gastrointestinal tract. During the cloning of Gal-4 cDNA from mouse colon, a closely related cDNA was discovered and named Gal-6. Gal-6 shares 83% amino acid homology with Gal-4c, differing in a 24-amino acid region between the two CRDs. Studies specific to human Gal-6 have yet to be documented [36].

Galectin-11 (Gal-11) is a ruminant-specific galectin first reported in sheep, localized in the nuclei and cytoplasm of gastrointestinal and bile duct epithelial cells. It is also present in mucosa of infected animals' stomach and small intestine, regulating immune and inflammatory responses post parasitic infection. Gal-11, a prototype galectin, consists of a single CRD and exists as a dimer, binding to carbohydrates such as glycoproteins or glycolipids [37].

Galectin-15 (Gal-15) (also known as OVGAL11) was also discovered in the sheep uterus, initially proven to be induced in gastrointestinal tissues and secreted into the intestinal lumen to address inflammation and eosinophil infiltration following infection with twisted blood intestine bacilli in sheep. Gal-15 is a unique member of the galectin family found in the Artiodactyla order including sheep, goats, and cattle, with no related genes detected in the human genome. The amino acid sequence of Gal-15 shows high similarity to human Gal-10 (also known as CLC) and Gal-13 (also known as PP13), containing a conserved CRD that binds β-galactosides and a separate integrin-binding domain, cross-linking cell surface glycoproteins and glycolipids, initiating a series of biological responses. Initially called OVGAL11 due to its lack of known homologues, it was considered a new member of the galectin family, later renamed Gal-15 [38].

This review primarily discusses the structural function of human galectins and their roles in diseases. Galectins not clearly present in human tissues, such as Gal-5, −6, −11, −15, will not be discussed in detail.

#### Galectin-12

Identified in 2001, Galectin-12 possesses two CRDs connected by a peptide chain. The C-CRD of Galectin-12 notably differs from other galectin family members in the critical conserved amino acid residues essential for binding β-galactosides [39]. The expression of Gal-12 is tightly regulated. Its translation initiation codon is a weaker initiation site, and the mRNA encoding Gal-12 contains five AU-rich motifs (AUUA) in the 3’-UTR region, increasing mRNA instability and thus limiting the expression of Gal-12. Gal-12 is mainly expressed in adipose tissue and neutrophils, while its expression is also observed at low levels in the heart, pancreas, spleen, thymus, and peripheral blood leukocytes [40].

#### Galectin-13/14

Galectin-13 (Gal-13) and Galectin-14 (Gal-14) are believed to induce apoptosis in activated T cells and help mediate immune tolerance between maternal and fetal tissues. Gal-13 was originally identified and purified from the placenta. The encoding gene, *LGALS13*, is located on chromosome 19q13.1, closely neighboring the genes encoding other galectin members such as Gal, −7, −10, and −14 (Fig. 1B). The *LGALS13* gene features five major upstream promoter regions and four exons, with exon 4 and part of exon 3 encoding the CRD. Gal-13 is a unique prototype galectin and a homodimer with a molecular weight of 32 kDa that does not bind to β-galactosides. The dimer is stabilized by disulfide bonds between cysteine residues Cys136 and Cys138 at the C-terminus of each monomer. In the cytoplasm's reductive environment, these disulfide bonds cannot be formed between Gal-13 monomers [41]. Similar to other galectin family members, Gal-13 is synthesized in the cytoplasm without a signal peptide and then exported to the ECM through a non-classical transport mechanism. Predominantly expressed in the syncytiotrophoblast, amnion, and fetal endothelium, human Gal-13 is secreted into maternal circulation, where it can induce T cell apoptosis, remodel spiral arteries, and aid in trophoblast invasion of the decidua. Low levels of Gal-13 in maternal serum may lead to preeclampsia. It is also present in the spleen, kidneys, bladder, and is expressed in conditions such as hepatocellular carcinoma, neurogenic tumors, and malignant melanoma [42].

Gal-14 is a newly identified prototype galectin, showing 78% sequence similarity to Gal-13 [43]. Gal-14 was initially isolated from tissue in the fetal brain, with its encoding gene, *LGALS14*, located on human chromosome 19q13.2. Studies also indicate its expression in the placenta and less so in other tissues. To date, there are few studies on human Gal-14. However, high-resolution crystallographic studies of Gal-14 reveal it can form stable dimers. Each monomer of Gal-14 retains the classic "jellyroll" fold. However, the dimeric structure differs significantly, forming through interactions between β-strands S5 and S6 rather than through the N and C termini or F-face. Notably, Asn65, Trp72, and Glu75 are highly conserved residues in both chains. The presence of conserved residues is crucial for lactose binding [43, 44].

#### Galectin-16

The *LGALS16* gene, encoding Galectin-16 (Gal-16), is located on chromosome 19q13.2 (Fig. 1B). It comprises four exons and is part of a gene cluster on chromosome 19 that also encodes Gal-10, Gal-13, and Gal-14. This gene cluster's diversity and evolutionary origins are believed to be closely linked to placental development. Transposable long interspersed nuclear elements (LINEs) within this gene cluster are typically located at the boundaries of large inversions and duplicated gene units. These elements are associated with the ability of anthropoid primates to maintain highly invasive placental formation and placental phenotype variability, resulting in longer gestation periods and larger brain-to-body ratios in their offspring [45]. Gal-16 is a monomeric protein composed of 142 amino acids, characterized by the typical β-sandwich structure of galectins. It consists of two domains formed by six β-strands (S1-S6) on the concave side and five β-strands (F1-F5) on the convex side.

### Expression, distribution, localization

Galectins are found in numerous human tissues such as the placenta, gastrointestinal tract, lungs, spleen, and heart, and are present in epithelial cells, endothelial cells, neurons, and immune cells. Each cell type expresses at least one form of galectin, with the specific type of galectin expressed varying depending on the cell's state [46]. After synthesis in the cytoplasm, galectins are transported to different subcellular locations. Galectins do not possess the signal peptide sequence necessary for the secretion of proteins, yet some galectins, such as Gal-1 and −2, are still secreted proteins. These galectins are produced within the cytoplasm and then released into the extracellular space, relying on their lectin activity to perform extracellular functions. Galectins are secreted into the extracellular environment through a non-standard pathway, bypassing the classic endoplasmic reticulum to Golgi transport system. They are mainly transported via vesicles and exosomes, then deposited in the ECM or attached to the cell membrane. This unique mode of secretion also prevents premature adhesion of galectins to glycoprotein oligosaccharides after synthesis.

Gal-1 can be found in both the nucleus and cytoplasm. It can also move to the inner surface or outside of the cell membrane, showcasing dual roles inside and outside the cell [47]. Gal-1 exhibits typical cytoplasmic protein characteristics, including an acetylated N-terminus, and lacks glycosylation modifications [48]. Gal-1 is found in the ECM of both normal and cancerous tissues and is secreted by multiple matrix components including fibroblasts, endothelial cells, macrophages, dendritic cells, and T cells. Its expression level within the tumor matrix might be closely linked to tumor stage and patient prognosis [49]. Gal-1 utilizes a non-classical, inside-out secretion pathway involving direct translocation across the cytoplasmic membrane. This process requires assistance from unspecified integral membrane proteins and cytoplasmic factors. Surface molecules containing β-galactoside sequences may act as receptors for Gal-1 secretion. Additionally, sodium pumps (Na + /K + -ATPases) likely play a significant role in its secretion process [50]. Gal-1 is variably expressed in a range of normal and pathological tissues, demonstrating diverse biological functions [47].

Like Gal-1, Gal-3 is extensively expressed across human tissues. It is found in various immune cells, epithelial and endothelial cells, as well as neurons. During embryonic development, Gal-3 expression is more abundant and specific compared to adulthood, primarily concentrated in epithelial tissues, cartilage, kidneys, and liver [51]. Gal-3 is distributed across the cell, appearing in the nucleus, cytoplasm, and on the cell surface, and it can also be secreted into the ECM. It is mostly located in the cytoplasm and moves between the cytoplasm and nucleus. The N-terminal region with the phosphorylated Ser6 site is essential for nuclear transport, and mutations here can hinder Gal-3's movement from the nucleus to the cytoplasm [11]. Other key sequences for Gal-3 localization include the CRD, which houses nuclear localization sequences (NLS) and nuclear export sequences (NES). These sequences enable Gal-3 to interact with the nuclear import complex Importin α and the nuclear pore protein Nup98, facilitating nucleocytoplasmic shuttling in both directions and aiding the cell in stress resistance [52–55]. During nuclear transport, monomeric Gal-3 usually enters the nucleus by passive diffusion, while polymeric Gal-3 requires an import mechanism [56]. Gal-3 does not have the traditional signal sequences needed to guide proteins to the endoplasmic reticulum and Golgi apparatus for secretion. It can be secreted through a non-classical pathway possibly involving vesicles, autophagic vesicles, and exosomes, allowing Gal-3 to enter biological fluids such as serum, urine, and saliva [57, 58]. The functions associated with Gal-3’s different subcellular localizations vary. In the cytoplasm, Gal-3 interacts with various proteins related to cell survival, such as Bcl-2 and K-Ras. Within the nucleus, Gal-3 influences gene transcription by impacting mRNA splicing. Extracellularly, Gal-3 primarily controls interactions between cells and with the ECM [59]. Given its widespread distribution and complex interactions, Gal-3 is involved in regulating many biological functions, including cell proliferation, apoptosis, differentiation, transformation, angiogenesis, inflammation, fibrosis, and host immune defense.

The expression of Gal-3 in tumor tissues is complex and controversial. It is significantly upregulated in highly underdeveloped and early aggressive colon cancers and is upregulated in the serum and tumor tissues of renal cell carcinoma patients [60]. In some types of thyroid cancer, such as papillary thyroid carcinoma (PTC), Gal-3 may serve as a potential immunomarker, with positive expression linked to lymph node metastasis [61]. In contrast, for other thyroid cancer types, the level of Gal-3 expression does not show a significant correlation with factors such as extrathyroidal extension, lymph node metastasis, overall metastasis, age, completeness of surgical resection, tumor aggressiveness, or size score [62]. Gal-3 contributes to the advancement of hepatocellular carcinoma (HCC) and stimulates tumor angiogenesis. Increased serum levels of Gal-3 are linked to poor prognosis and metastasis to the portal vein in HCC patients. However, there is no significant difference in serum Gal-3 levels between patients with HCC and those with cirrhosis [63, 64]. Gal-3 expression on the surface of HCC cells may be useful for prognostic purposes, but its utility for diagnostic purposes in HCC is limited. In breast cancer tissues, Gal-3 expression levels are significantly higher compared to adjacent non-tumor tissues [65]. However, certain studies have indicated that reduced Gal-3 expression in breast cancer is significantly linked to enhanced tumor vascular infiltration and decreased survival rates [66]. Numerous studies have explored the expression and significance of Gal-3 in various cancers, including prostate, pancreatic, bladder, gastric cancers, and lymphoma. Overall, Gal-3 expression is abnormal in various types of tumors, but there is no conclusive evidence to support Gal-3 as a tumor-specific marker, and it should be evaluated in conjunction with other specific markers.

Unlike Gal-1 and Gal-3, which have widespread expression patterns, other family members exhibit higher tissue specificity. Gal-2 is confined to the epithelium of the gastrointestinal tract. In contrast, Gal-4 is expressed exclusively in the epithelial cells of the digestive tract, spanning from the tongue to the colon, including the gastric antrum, ileum, colon, and rectum [67]. Gal-4 is downregulated in colon cancer tissues but significantly elevated in the serum of individuals with colon and breast cancer. It is also upregulated in pancreatic, liver, and stomach cancer tissues [20, 60, 68]. Gal-4 is mainly found in the apical extracellular region of intestinal cells and is involved in transporting proteins to the apical surface of intestinal epithelial cells [69].

Gal-7 is specifically expressed by keratinocytes and is found in all layers of the epidermis and stratified epithelial tissues, most notably in the tongue, esophagus, cornea, stomach, anus, and the Hassall's corpuscles of the thymus [70–73]. Subsequent studies have confirmed that other types of epithelial cells, such as myoepithelial cells in the mammary gland, also express Gal-7 [74]. Gal-7 expression fluctuates according to the degree of stratified epithelial differentiation and appears to be an indicator of various differentiation stages in keratinocytes. Gal-7 might contribute to the formation and maintenance of stratified epithelium by modulating cell proliferation and intercellular interactions [70, 73]. Although most studies focus on the expression of Gal-7 in epithelial-derived cells, it is also present in cells of other lineages, often overlooked due to its low levels and thus potentially lost in large-scale data analysis. Gal-7 expression can be induced by p53 and ultraviolet B (UVB) [75]. Similar to other galectins, Gal-7 lacks a conventional secretion signal peptide. Normally, Gal-7 is found in the cytoplasm, nucleus, and mitochondria. A mutation at the 74th amino acid can affect its translocation to mitochondria and the nucleus and its ability to bind to glycoproteins [22]. The intracellular role of Gal-7 is mainly associated with the proliferation and differentiation of keratinocytes and can be modulated by the c-Jun N-terminal kinase (JNK1) [76]. The functions of Gal-7 in the nucleus are not clear, but p53 and nuclear factor κB (NF-κB) can bind to the Gal-7 promoter and regulate its activation [77].

Gal-8 is a protein broadly expressed across numerous organs and tissues, present in both normal and disease states, such as in inflamed synovium, joint areas, and tumor tissues [78]. It is found in both primary lymphoid organs, such as the bone marrow and thymus, and secondary lymphoid organs, including the spleen and lymph nodes, where it helps regulate innate and adaptive immunity [78]. Gal-8 is a key regulator of primary tumor growth and metastasis, with significant expression in lung cancer tissues as well as in breast and prostate tumors [79–82]. Gal-8 is located in the cytoplasm, cell membrane, and nucleus, and it is released into the extracellular environment through a non-classical pathway because it lacks an N-terminal signal peptide sequence [83].

Gal-9 was first discovered in human Hodgkin's lymphoma tissues and in the embryonic kidneys of mice [84, 85]. It is primarily distributed in the thymus, liver, intestines, kidneys, spleen, lungs, myocardium, skeletal muscle, brain, pancreas, bladder, prostate, and placenta. Gal-9 induces apoptosis in T cells during negative selection, regulating the development of the acquired immune system and is also expressed in other leukocytes responsible for innate and acquired immunity, activated endothelial cells, and IFN-stimulated fibroblasts [27, 85]. In tumor cells, the expression of Gal-9 differs by tumor type, showing reduced levels in hepatocellular carcinoma, prostate cancer, colorectal cancer, cervical cancer, and skin cancer [86–89]. In oral cancer, pancreatic cancer, polymorphic glioblastoma, and hematologic malignancies, Gal-9 expression is increased compared to adjacent non-tumor tissues [84, 90, 91].

Primarily located in the cytosol and also present in the nucleus, Gal-9 has an unclear secretion mechanism. Without hydrophobic signal peptides, it does not access the endoplasmic reticulum and thus cannot be secreted through the traditional pathway to the extracellular environment. It may instead be released via non-classical pathways such as direct translocation through the cytoplasmic membrane or release into exosomes or lysosomes, and microvesicles [92]. In the cytoplasm, Gal-9 plays a role in regulating numerous cellular functions, such as apoptosis and cell aggregation, although its nuclear role is still not well understood. Extracellularly, Gal-9 can attach to specific glycoproteins or glycolipids in the ECM or on cell surfaces, typically interacting with proteins like T-cell immunoglobulin and mucin domain-containing protein 3 (Tim-3) and protein disulfide isomerases, among others [93, 94], mediating chemotaxis, cell adhesion and migration, receptor endocytosis, raft clustering, and lipid cycling [95].

Gal-10 mRNA is extensively transcribed in bone marrow, potentially playing a crucial role in the maturation of lymphocytes [31]. Highly expressed in eosinophils and also found in T cells, Gal-10 on eosinophils forms synapses with the T cell membrane [96]. Gal-10 can inhibit the function of CD25 + Treg cells [97]. In individuals with atopic dermatitis, elevated levels of Gal-10 are observed in CD3 + T cells [98]. Gal-10 is found in nuclear chromatin, cell membrane fragments, and phagosomes. A significant amount of Gal-10 is concentrated in the nuclei of eosinophils [30]. Numerous reports indicate that Gal-10 is found in the cytoplasm as well as in both intracellular and extracellular membrane regions. Gal-10 can attach to T cell membranes, thereby suppressing their function. Upon stimulation by triggers or during apoptosis or necrosis, eosinophils undergo ETosis, rapidly disintegrating the plasma membrane and releasing total cellular contents, releasing Gal-10 into the extracellular space [32].

Gal-12 is expressed in adipocytes and macrophages, acting as an intrinsic negative regulator of lipolysis, and can be upregulated by hypoxia [40]. It is also found at low levels in the pancreas, heart, spleen, thymus, and peripheral blood leukocytes [99]. Gal-12 expression is notably reduced in several cancers, such as acute lymphoblastic leukemia, colon cancer, and prostate cancer, suggesting it may function as a tumor suppressor [100]. Gal-12 is localized in the nucleus, but the mechanism of its shuttling between the nucleus and cytoplasm remains unclear.

Gal-13 and Gal-14 are important effector molecules in embryology, and their dysregulated expression is involved in inducing pre-eclampsia [41]. Gal-13, exclusive to primates, was initially identified and purified from the placenta. Human Gal-13 is primarily expressed in the syncytiotrophoblast, placenta, fetal endothelium, and also found in the kidneys, spleen, and bladder. It is also expressed in hepatic adenomas, neurogenic tumors, and malignant melanomas [42]. Initially isolated from fetal brain tissue, Gal-14 is predominantly expressed in the placenta, where its levels surpass those of other galectins. It plays a crucial regulatory role in fetal development and immune tolerance during pregnancy [43]. Gal-13 and Gal-14 are thought to regulate the apoptosis of T cells and other leukocytes, thus regulating immune tolerance between the mother and fetus.

Gal-16 is specifically overexpressed in the placenta and also overexpressed in brain tissues and the retina, but experimental studies on Gal-16 are very limited, and the biological significance and reasons for its overexpression in brain tissues and the retina remain unclear [45].

## The binding partners and signal cascade of galectins

Galectins were initially described as lectins that bind to β-galactosides, with each family member possessing an evolutionarily conserved CRD capable of recognizing β-galactosides, especially polysaccharides containing LacNAc. The function of galectins depends not only on the molecule itself but also significantly on its specific binding partners. Except for a few proteins, most cell surface receptors and secreted proteins are highly glycosylated, representing a highly regulatable post-translational modification. Variations in glycosylation affect the recognition of glycosylated proteins by galectin-mediated glycan-binding proteins, forming a highly complex and precise regulatory network. Galectins attach to glycoconjugates on cell surfaces, including proteins and lipids, facilitating cell communication by mediating interactions between cells or between cells and extracellular elements. Most galectins not only possess lectin activity that binds to β-galactosides but also engage in protein–protein interactions, broadly regulating various cellular signaling pathways, including oncogenic signals.

### Galectin-1 binding partners

#### Binding with carbohydrates

The CRD domain of Gal-1 binds to LacNAc residues on cell surface receptors and extracellular glycoproteins, including integrins, CD43, CD45, fibronectin, mucins, laminin, platelet activation proteins, vitronectin, and osteopontin [49]. Dimeric Gal-1 (dGal-1) preferentially binds to disaccharides containing N-acetyllactosamine (Gal-β1–3/4 GlcNAc, also known as LacNAcII or type 2 sugar), and shows lower affinity to glycoproteins with a single galactose unit. dGal-1 can establish surface lattices on cell membranes with various glycoproteins, mediating cell surface signaling or regulating endocytosis. Most structural studies of Gal-1 report its binding to simple carbohydrates; however, this is not the whole story. Gal-1 can interact with more complex glycans of varying sizes and compositions, thereby exerting its functions on the cell surface [101].

Integrins are crucial for the survival and proper function of both normal and cancerous cells. dGal-1 directly binds to β1 integrin, increasing the activation of a subset of β1 integrin molecules. In vascular smooth muscle cells, Gal-1 interacts with α1β1 integrin, enhancing focal adhesion kinase (FAK) phosphorylation, thereby regulating cell adhesion, migration, and spreading [102]. In epithelial cancer cells, Gal-1 interacts with integrin α5β1, inhibiting cell growth. The interaction of dGal-1 with integrin receptors controls adhesion of atypical tumor cells, recognizing glycans on ECM proteins such as laminin or fibronectin to regulate cell migration and invasion. Additionally, Gal-1 plays a role in the assembly and remodeling of the ECM, preventing the integration of thrombospondin and sulfated chondroitin B into the ECM of vascular smooth muscle cells [47]. Gal-1 specifically binds to several surface glycoproteins on T cells in a carbohydrate-dependent manner, including CD2, CD3, CD7, CD43, and CD45, regulating the survival of immune and inflammatory cells [47].

On the surface of human neuroblastoma cells, Gal-1 serves as a primary receptor for the carbohydrate segment of ganglioside GM1. Cell fusion increases surface expression of dGal-1, which negatively regulates neuroblastoma cell growth without inducing apoptosis (Table 1).

**Table 1:** Binding partners of galectins

Galectin	Binding partner	Cell type	Associated processes
Galectin-1	α1β1 integrin	Vascular smooth muscle cell and tumor cells	Cell adhesion and migration diffusion
	α5β1 integrin	Tumor cells	Tumor suppression
	α4 integrin	Pre-B cells	Signaling and maturation
	CD2	T cells	Apoptosis
	CD3	T cells	Apoptosis
	CD7	T cells and thymocytes	Apoptosis
	CD43	T cells and thymocytes	Apoptosis and transendothelial migration
	CD45	T cells and thymocytes	Apoptosis
	TCR	T cells	Signaling and activation
	Pre-BCR	Pre-B cells	Signaling and maturation
	Neuropilin 1	Endothelial cells	Migration
	GM1	T cells and Human neuroblastoma	Treg cell-mediated immunosuppression and Tumor suppression
	Ras	Tumor cells	Cell transformation and anti-proliferation
	Gemin4	Tumor cells	preRNA splicing, RNA interference
	Protocadherin-24	Tumor cells	Tumor suppression
Galectin-2	CD29	T cells	Apoptosis
	GM1	Human neuroblastoma	Growth-regulatory interaction
	MUC1	Colon cancer and breast cancer	Promote cancer cell adhesion
	MUC5AC	Gastric mucus	Protect the mucosal surface
	β1 integrin	T cells	Apoptosis
	Lymphotoxin-α (LTA)	Macrophages and atherosclerotic plaque	Myocardial infarction
Galectin-3	α1β1、α3β1、α4β7、α6β1 and αMβ1	Tumor cells	Cell migration
	N-cadherin	Breast cancer	Cell migration
	Bcl-2	Tumor cells	Anti-apoptotic
	K-Ras	Tumor cells	Regulating gene expression and anti-apoptotic
	ANXA7	-	Anti-apoptotic
	CD7	T cells and thymocytes	Apoptosis
	CD29	T cells	Apoptosis
	CD45	T cells and thymocytes	Apoptosis
	CD71	T cells	Apoptosis
	CD98	Macrophages	Alternative activation
	TCR	T cells	Signaling and activation
	CTLA4	T cells	Cell growth arrest
	LAG3	T cells	Activation of CD8 + T
	LGALS3BP	Tumor cells	Cancer progression
	Fascin-1	Tumor cells	Tumor metastasis
	GSK-3b	Tumor cells	Tumor metastasis
	AP-1	Tumor cells	Tumor metastasis
	Neogenin-1	Tumor cells	Tumor metastasis
	C/EBPb	Tumor cells	Cancer progression
	EGF and bFGF	Tumor cells	Cancer cell stemness
Galectin-4	GM1	Neuroblastoma	Growth regulation
	MUC1	Tumor cells	Carcinogenic signals
	Tn	Tumor cells	Carcinogenic signals
	CD3ε/δ	T cells	Apoptosis
	CD44	Tumor cells	Anti-proliferation and anti-migration
	c-Met	Tumor cells	Anti-proliferation and anti-migration
Galectin-7	Bcl-2	Tumor cells	Anti-apoptotic
	E-cadherin	Tumor cells	Tumor metastasis
	Tid1	Tumor cells	Cancer progression
Galectin-8	β1, αM, α3β1, α6β1 integrin	-	Signaling and activation
	α4 integrin	Pre-B cells	Signaling and maturation
	CD44	Synovial fluid cells	Apoptosis
	NDP52	-	Autophagy
	LILRB4	M-MDSC	Tumor immune suppression
	PDPN	TAMs	Angiogenesis and lymphatic invasion
	K-Ras4B with Farnesylation	Tumor cells	Signaling and activation
	NUFIP2	HEK293T/Tumor cells	Protein synthesis inhibition
Galectin-9	Tim-3	TH1 cells	Apoptosis
		Dendritic cells and monocytes	Maturation and cytokine secretion
	TLR-4	Microglia	Anti-inflammatory
	VISTA	T cells	Apoptosis
	Dectin-1	Macrophage	Immune tolerance reprogramming and adaptive immune suppression
	CD44	T cells	Adhesion
	4-1BB(CD137)	T cells/NK cells/DC	Function
	DR3	Treg	Anti-inflammatory
	IgE	B cells	Anti-allergy
	IgM	B cells	Signaling
	BCR	B cells	Signaling
	LMP-1	Tumor cells	Cancer progression
	Glut-2	Pancreas β cell	Insulin secretion

#### Binding with proteins

Gal-1 is capable of interacting with proteins without relying on its carbohydrate-binding abilities, and these proteins lack shared structural domains or motifs. One particularly intriguing and potentially important function of Gal-1 is its interaction with Ras, which occurs both inside and outside the cell, each modulating different effects—promoting cellular transformation and exerting anti-proliferative actions. In human cancers, frequent mutations in Ras promote malignant transformation. Intracellular Gal-1 stabilizes activated H-Ras (G12V) on the plasma membrane in a lactose-independent manner, interacting with H-Ras-GTP to induce oncogenic Ras signaling pathways. H-Ras-GTP recruits Gal-1 from the cytosol to the cell membrane, where it aggregates in non-raft microdomains and subsequently binds to Raf-1 to activate the ERK signaling pathway, promoting cellular transformation [103]. Extracellular dGal-1 binds with integrin α5β1, suppressing the Ras-MEK-ERK pathway, promoting p21 transcription, and increasing p27 levels. It inhibits cyclin-dependent kinase 2 activity, causing G1 phase arrest and hindering growth, thereby exerting anti-proliferative effects on cancer cells [104]. In the cell nucleus, Gal-1 interacts with Gemin4 and co-immunoprecipitates with the nuclear SMN complex, facilitating pre-mRNA splicing [105]. Gal-1 also interacts with Protocadherin-24 on the cell membrane, where it is retained and inhibits β-catenin signaling through localization via β-catenin [46]. Extracellularly, Gal-1 primarily exhibits lectin activity, while its protein–protein interactions are mainly associated with its intracellular functions.

Gal-1 is involved in the regulation of different immune cells, significantly affecting B cell differentiation in the bone marrow, with a special emphasis on pre-BII cell development. The expression of the pre-B cell receptor (pre-BCR) triggers essential checkpoints in B cell maturation. Gal-1 functions as a ligand for the pre-BCR by directly interacting with its surrogate light chain λ5, leading to receptor clustering. This process promotes the proliferation and differentiation of pre-BII cells [106].

### Galectin-2 binding partners

PolyLacNAc is a common ligand for galectins, and Gal-2 has a stronger binding capacity for complex oligosaccharides with terminal galactose than for simple monosaccharides or disaccharides containing galactose [107]. The 3-O-sulfation of the galactose residues in LacNAc enhances Gal-2 binding. Many cellular glycoproteins bind with Gal-2, such as ganglioside GM1 in neuroblastoma cells [108], mucin protein MUC1 in colon and breast cancer [60], and glycoprotein MUC5AC in gastric mucus [109]. Gal-2 binds to T cells in a carbohydrate-dependent manner, distinguishing itself from Gal-1 by its lack of binding to CD3 and CD7. Instead, Gal-2 targets β1 integrin for interaction with T cells [110]. The presence of lactose can reduce their binding. Gal-2 is also implicated as a risk factor in myocardial infarction, where it interacts with the cytokine lymphotoxin-α (LTA) in macrophages following myocardial infarction and in atherosclerotic plaques [111]. Gal-2 further mediates agglutination of red blood cells through binding to blood group-related glycans (Table 1).

### Galectin-3 binding partners

Gal-3 is ubiquitously expressed both intracellularly and extracellularly, demonstrating diverse functions through its interactions with various molecules across different subcellular localizations. Gal-3 binds to both type 1 and type 2 Galβ1–3(4)GlcNAc (N-acetyllactosamine) chains, exhibiting a higher affinity for poly-N-acetyllactosamine structures and branched polysaccharides. Gal-3, inside and outside of cells, attaches to glycoconjugates in the ECM, including laminin and fibronectin, along with mucins, elastin, type IV collagen, and tenascins-C and -R [112]. The interactions of Gal-3 with these ligand molecules can stimulate tumor cell migration and angiogenesis. Cell adhesion molecules like integrins also serve as receptors for Gal-3, including integrins α1β1, α4β7, α6β1, and αMβ1. The binding of Gal-3 with integrins relies on glycosylation. Gal-3 interacts with the N-glycosylation sites of integrin α3β1, activating key regulators of the integrin signaling pathway such as FAK, influencing the Rac1 protein involved in reorganizing the actin cytoskeleton and forming lamellipodia, thus affecting the cell’s migration capability [113]. In mouse breast cancer cells, when N-cadherin binds to extracellular Gal-3, cell–cell adhesion is disrupted, promoting tumor cell migration from the primary site.

Gal-3 plays a vital role in cell survival by interacting with proteins related to survival, such as Bcl-2 and K-Ras bound to activated GTP. It forms lectin lattices with cell surface glycoproteins and the ECM, acting as a scaffold for cellular signaling transductions and activating K-Ras-associated pathways. The interaction between Gal-3 and K-Ras can trigger downstream effectors like Raf and PI3K, which subsequently regulate gene expression. The NWGR motif of Gal-3 is highly homologous to the anti-apoptotic Bcl-2 protein. In vitro, Gal-3 interacts with Bcl-2 to form heterodimers, mimicking Bcl-2’s function. In various cancer cells, the Gal-3 and Bcl-2 interaction influences mitochondrial apoptosis, providing resistance to agents that induce apoptosis [114]. In Gal-3's anti-apoptotic function, it directly binds with calcium and the phospholipid-binding protein Synexin (Annexin A7). Additionally, the interaction between Gal-3 and K-Ras contributes to the regulation of its anti-apoptotic activity [115].

Immune effector cells identify specific surface proteins on tumor cells, initiating damage and hindering tumor growth. Nonetheless, aggressive tumor cells can release various cytokines and chemokines to disrupt immune cell function and escape immune detection. Tumor cells express and release Gal-3, which can interact extracellularly with CD7 and CD29 on T cells, leading to T cell apoptosis [116]. The interaction of Gal-3 with CD45 and CD71 also results in the death of T cells [117]. The T cell receptor (TCR) also binds with Gal-3, promoting TCR downregulation, affecting the receptor's activity and function [118]. Gal-3 serves as a negative regulator of T cell activation and can be guided to the cytoplasmic side of the immune synapse within the peripheral supramolecular activation cluster (pSMAC). Gal-3 disrupts the formation of IS and interacts with ALG-2 interacting protein X (Alix) in a sugar-independent manner to promote TCR downregulation [118]. Gal-3 can bind to lymphocyte activation gene 3 (LAG3), a transmembrane protein of the immunoglobulin superfamily, essential for activating CD8 + T cells. The binding of Gal-3 to LAG3 inhibits the interaction of MHC class II and LSECtin with LAG3 [119].

Lectin-galactoside binding soluble 3-binding protein (LGALS3BP, also called Gal-3BP, 90 K, Mac2BP) is a secreted multifunctional glycoprotein found in human influenza virus, serving as a new ligand for Gal-3 that drives cancer progression and dissemination [120]. LGALS3BP is highly glycosylated and is crucial for galectin-mediated biological processes. Fascin-1, a protein associated with cell movement, acts as an actin-bundling agent that induces membrane protrusions to promote tumor cell motility, highly expressed in various cancers such as gastric and lung cancer [121]. Gal-3 has a strong interaction with the Wnt pathway signaling molecule GSK-3b, affecting β-catenin and Fascin-1, influencing tumor metastasis [122]. Protease-activated receptor-1 (PAR-1) and matrix metalloproteinase (MMP)−1 are two proteins associated with cancer progression and motility. Upon activation, the cell surface receptor PAR-1 can undergo autophosphorylation, triggering matrix metalloproteinase-1 (MMP-1) and downstream signaling. The transcription factor complex AP-1, formed by c-Jun and Fra-1, regulates the transcriptional expression of PAR-1 and MMP-1. Gal-3 can directly interact with AP-1 to regulate the expression levels of PAR-1 and MMP-1 [123]. There are also reports that the interaction between Gal-3 and AP-1 regulates the expression of MUC2, impacting tumor progression and the motility of cancer cells [124]. Neogenin-1, a transmembrane receptor in the immunoglobulin superfamily, enhances tumor cell proliferation and movement. Its regulation is controlled by heat shock factor-1 (HSF-1). Gal-3 can directly bind to HSF-1, inducing its accumulation in the nucleus and upregulating the expression of Neogenin-1.

Gal-3 interacts with various transcription factors within the nucleus to regulate cancer progression and metastasis. The transcription factor CCAAT/enhancer-binding protein beta (C/EBPb) interacts with Gal-3 and LGALS3BP, promoting tumor progression. C/EBPb arbitrarily regulates the expression of the hyaluronic acid-mediated motility receptor (HMMR). HMMR interacts with hyaluronic acid on the cell surface, triggering a signaling cascade that activates tyrosine phosphorylation of intracellular signaling molecules. HMMR directly interacts with cytoskeletal proteins such as microtubules and spindles, regulating the motility of cancer cells [125]. These findings suggest that Gal-3's involvement in regulating tumor cell motility is multifaceted.

Cancer stem cells (CSCs) exhibit self-renewal capabilities and can undergo epithelial-to-mesenchymal transition, indicating a greater level of malignancy. Gal-3 interacts with various factors to regulate the stemness of cancer. Gal-3 binds to stem cell culture components EGF and bFGF, controlling the expression of the stem cell-associated transcription factor KLF4. The interactions of Gal-3 with integrin αvβ3, K-Ras, and Wnt pathway signaling molecules are also crucial for the stemness of cancer cells (Table 1) [11].

### Galectin-4 binding partners

Gal-4 interacts with ligands through ionic bonds and van der Waals forces. In the realm of glycolipids, Gal-4 chiefly interacts with sulfated types, such as gangliosides like GM1, cholesterol-3-sulfate, and other similar molecules [21]. Gal-4 exhibits a stronger affinity for O-glycans compared to N-glycans. Its interaction with ligands mediates its primary physiological functions. Mucin 1 (MUC1), a glycoprotein that spans the membrane, naturally binds to Gal-4, often aberrantly glycosylated and overexpressed in various epithelial cancers. It shows highly sialylated forms in breast cancer and colon cancer cells [126]. Another form of MUC1, with low glycosylation, accumulates intracellularly, triggering oncogenic signals. Thomsen-nouveau (Tn) antigen is another natural ligand for Gal-4, overexpressed in poor prognosis breast cancer, gastric cancer, and colorectal cancer [127].

Gal-4 has a dual function in regulating the immune system: it can increase IL-6 secretion by CD4 + T cells, exacerbating inflammatory responses, while also binding to N-glycosylated residues on the CD3ε/δ surface receptors of T cells, promoting tumor immune tolerance and inducing apoptosis in T cells. Gal-4 has been found to bind to cell membrane proteins CD44 and c-MET through its carbohydrate-binding ability. Knocking down Gal-4 activates CD44 and c-MET, reducing the potential for the growth and spread of malignant gastric cancer cells to the peritoneum (Table 1) [128].

### Galectin-7 binding partners

Gal-7, similar to other galectins, engages with various potential receptors, such as internal LacNac oligosaccharide residues and non-reducing terminal LacNac residues [22]. While the reported non-carbohydrate binding ligands for Gal-7 are fewer compared to Gal-1 and Gal-3, it is undeniable that Gal-7 also interacts with non-glycan proteins in a manner independent of its CRD domain, thereby participating in the regulation of cellular functions.

Using mitochondrial proteomics approaches, endogenous Bcl-2 has been found to co-immunoprecipitate with Gal-7 during investigations into a potential new cooperative network. Recombinant Bcl-2 and Gal-7 can directly bind in vitro, independent of Gal-7's CRD. Some Gal-7 proteins localize to mitochondria in a Bcl-2-dependent manner, sensitizing mitochondria to apoptotic signals [129]. Gal-7 inhibits soluble galectin expression in epithelial cells, regulating cell migration, intercellular adhesion, and associated cancer cell invasion behavior, and induces matrix metalloproteinase MMP-9 expression. E-cadherin is a well-known substrate of MMPs, including MMP-9, and studies have shown that Gal-7 is a direct binding partner of E-cadherin, binding to its extracellular domain independently of the Gal-7 CRD domain. Addition of LacNac does not disrupt their binding. This interaction stabilizes E-cadherin on the plasma membrane, inhibiting its endocytosis [130]. Heat shock protein tumorous imaginal disc (Tid1), known to reduce head and neck squamous cell carcinoma (HNSCC), interacts with Gal-7 via N-linked glycosylation bridges. Low Tid1 and high Gal-7 levels predict poorer survival rates in HNSCC patients (Table 1) [131].

### Galectin-8 binding partners

As an extracellular ligand, Gal-8 has dual recognition abilities, activating signaling pathways via both protein-carbohydrate and protein–protein interactions. Gal-8 interacts with the receptor for autophagy cargo NDP52 via protein–protein interaction. NDP52 binds to the convex surface of Gal-8's CRD, which does not interfere with lactose binding on the concave surface, allowing Gal-8 to simultaneously bind to proteins and carbohydrates [132].

Gal-8 has a high-affinity functional ligand known as Leukocyte immunoglobulin-like receptor B4 (LILRB4), which acts as an immune inhibitory receptor. Their interaction activates STAT3 and suppresses NF-κB, inducing M-MDSCs to promote immune suppression and tumor growth [133]. Gal-8 also binds receptor complexes including uPAR, MRC2, and LRP1, mediating cytokine expression. Integrins such as β1, αM, α3β1, and α6β1 are binding partners of Gal-8, and their complex formation triggers integrin-mediated signaling cascades, activating FAK, ERK, and PI3K pathways [26]. In infiltrating immune cells of breast tumors, transmembrane glycoprotein Podoplanin (PDPN) expressed on some tumor-associated macrophages (TAMs) is another binding partner of Gal-8. PDPN interacts with Gal-8 derived from lymphatic endothelial cells (LECs) in a glycosylation-dependent manner to promote activation of integrin β1, ultimately leading to local matrix remodeling, angiogenesis, and lymphatic invasion [134]. Studies have also documented direct interactions of Gal-1 and Gal-3 with H-Ras and K-Ras, respectively, activating Ras signaling. In contrast, Gal-8 binds to farnesylated K-Ras4B, inhibiting Ras activation [135].

Recent research has shown that Gal-8 localized to lysosomes also participates in widespread protein synthesis inhibition following lysosomal damage. Lysosomal damage leads to the formation of stress granules (SG) via EIF2A/eIF2α phosphorylation. Core SG proteins NUFIP2, G3BP1, and mATG8 family member GABARAP recruit to damaged lysosomes. On lysosomes, NUFIP2 collaborates with Gal-8 through the Ragulator RRAGA-RRAGB complex to promote MTOR inactivation, thereby controlling protein translation (Table 1) [136].

### Galectin-9 binding partners

#### Binding with carbohydrates

The binding strength of galectins to N-glycans relies on the valency of the glycan compound. Gal-9 displays strong affinity toward branched N-glycans and repetitive low-molecular-weight N-acetylglucosamine (GlcNAc) units. Significantly higher interaction strengths was observed with triantennary N-glycans compared to biantennary and monovalent N-glycans (Table 1).

#### Binding with proteins

Members of the galectin family are vital in maintaining immune cell balance and regulating inflammation, as they interact with various receptors in the immune system. Gal-9 engages with multiple cell surface receptors and ECM proteins. In reports, Gal-9 has been shown to directly interact with or bind to several surface partners, including T cell immunoglobulin domain and mucin domain-3 (Tim-3), PD-1, glucose transporter 2 (Glut-2), glucagon receptor, protein disulfide isomerase, Epstein-Barr virus latent membrane protein-1 (LMP-1), immunoglobulin E, adhesion molecule cluster of differentiation 44 (CD44), CD40, 4-1BB, DR3, V-domain Ig-containing suppressor of T cell activation (VISTA), and Toll-like receptor 4 (TLR-4). These interactions cover regulation of T cell development, maturity, and apoptosis, along with the modulation of immune cells like B lymphocytes and macrophages.

Tim-3 is a membrane protein specific to Th1 cells and an immune checkpoint receptor that balances innate and adaptive immunity. Tim-3 is absent on naive T cell surfaces but emerges following differentiation into Th1 cells. It plays a role in regulating Th1 immune responses and modulating macrophage activation, which in turn influences autoimmune diseases [137]. Tim-3 is a well-studied natural ligand of Gal-9. When naive T cells differentiate into Th1 cells, Gal-9 is expressed and serves as a ligand for Tim-3. This interaction causes Th1 cell aggregation and apoptosis through a Tim-3-dependent mechanism, thereby suppressing Th1 immune responses [137, 138]. The T cell receptor PD-1 interacts with Gal-9, shielding T cells expressing Tim-3 from apoptosis induced by Gal-9 [139]. The binding of Gal-9 to Tim-3 stimulates IL-12 production, initiating adaptive immune responses. This interaction depends on the CRD of Gal-9 interacting with the β-galactoside domain of Tim-3, and lactose addition can antagonize the binding between Gal-9 and Tim-3. Gal-9 plays a complex role in immune regulation, acting similarly to new immune checkpoint inhibitors following PD-1/PD-L1. Gal-9 also interacts with CD40, inhibiting the proliferation and survival of CD40 + effector T cells with low CD4 expression, this process induces cell death through a mechanism independent of Tim-3 [140].

TLR-4 is a classical molecular receptor involved in innate immunity. Gal-9 can bind to TLR-4 and participate in regulating central nervous system immune responses, promoting the transformation of microglia into the M2 phenotype and secretion of anti-inflammatory factors [141]. VISTA, present on T cells, identifies Gal-9 secreted by acute myeloid leukemia (AML) cells as its ligand. This recognition leads to changes in T cell membrane potential, activation of cytotoxic T cell granule enzyme B, induction of T cell apoptosis, and reduction in immune attack against tumor cells [142]. Dectin-1, an innate immune receptor, is abundantly expressed on macrophages within pancreatic ductal adenocarcinoma. Within the tumor microenvironment, Gal-9 attaches to Dectin-1 in a manner that does not depend on polysaccharides, leading to macrophage reprogramming towards immune tolerance and suppressing adaptive immunity. Inhibiting the Dectin-1-Gal-9 signaling pathway reprograms CD4 and CD8 T cells to promote anti-tumor immune responses [143]. CD44, a glycoprotein found on the surfaces of epithelial cells, plays a significant role in cell adhesion and interaction. Hyaluronic acid (HA) is its major ligand [144]. Gal-9 specifically binds to CD44 and negatively regulates CD44-HA interactions, reducing allergic inflammation in cases of chronic asthma. [145]. Gal-9 binding to CD44 promotes the differentiation and stability of adaptive regulatory T cells [146], and promotes Smad phosphorylation and osteoblast differentiation [147]. 4-1BB (CD137), part of the TNF receptor superfamily, functions as a co-stimulatory receptor for activated T cells, NK cells, and dendritic cells. Gal-9 binds to 4-1BB in a carbohydrate-dependent manner, promoting aggregation and functional activity of 4-1BB on T cells, NK cells, and DCs [148, 149]. Additionally, studies indicate that another TNFR family molecule, DR3, binds to the extracellular region of Gal-9, mediating expansion and activation of CD4^+^Foxp3^+^ Treg cells, and suppressing inflammation [150]. IgE, a highly glycosylated immunoglobulin, specifically binds to Gal-9, preventing formation of IgE antigen complexes and inhibiting excessive degranulation, thus exhibiting anti-allergic effects [151]. Gal-9 also binds to glycosylated proteins IgM and B cell receptor (BCR), regulating B cell signal transduction [152]. Nasopharyngeal carcinoma (NPC), linked to Epstein-Barr virus (EBV), produces Epstein-Barr virus latent membrane protein-1 (LMP-1). The transport and signaling of LMP-1 require raft infiltration. Gal-9 is abundantly present in NPC and acts as a specific partner for LMP-1 protein [153]. Present on the surface of pancreatic β cells, Glut-2 is vital for insulin secretion when glucose stimulates these cells. When Gal-9 binds to Glut-2, it prevents its internalization and keeps it on the cell membrane, supporting insulin production in response to glucose and aiding in glucose homeostasis (Table 1) [154].

### The binding properties of Gal-10, 13, 14, 16

Gal-10's natural ligands include both carbohydrates and proteins. It shows reduced affinity for agarose beads that are modified with N-acetyl-D-glucosamine and lactose. The crystal structures of Gal-10 demonstrate its capacity to bind mannose, yet it exhibits no affinity for β-galactosides. The specific ligands of Gal-10 in the nucleus and cytoplasm are not yet clear. Gal-10 has the ability to attach to unidentified glycans present on the membranes of T cells, resulting in downregulation of T cell functions (Table 1) [30].

Gal-13, Gal-14, and Gal-16 are predominantly present at the maternal–fetal interface and might trigger apoptosis in activated T cells. However, their interaction partners on immune cells and their regulation of signaling pathways remain undisclosed. Distinct from other galectins, Gal-13, −14, and −16 are unable to bind β-galactosides. Wild-type Gal-13 cannot associate with lactose; it only binds lactose if Arg53 and His57 are mutated to His53 and Arg57. Gal-14 also shows poor affinity for lactose, but replacing Arg55 with asparagine restores lactose binding capability. Gal-14 and Gal-16 can localize to the nucleus and engage with NF-κB family member c-Rel, thereby regulating lymphocyte activity (Table 1) [155].

## Pathological and physiological functions of galectins

The cell surface is abundant in glycosylated molecules, whose various glycosylation states can interact with carbohydrate-binding proteins, such as lectins, to mediate different signaling pathways [156]. Galectins, which can bind to β-galactosides, play a significant role in these interactions. Galectins can form lattices by cross-linking glycoproteins or glycolipids on the cell surface, thanks to their oligomerization potential and interaction with various glycosylated molecules. This cross-linking affects cellular activities, including adhesion, migration, signaling pathways, and survival of cells [157]. Alterations in protein glycosylation and galectin levels are strongly linked to the progression of numerous diseases, such as cancer. Aberrant expression of galectins is frequently associated with cancer initiation, progression, and metastasis, and they have extensive regulatory effects on immune cells [158].

### Cell proliferation

Gal-1 is extensively expressed across the body and plays a role in regulating cell proliferation, adhesion, differentiation, development, signaling pathways, and immune functions [159]. Gal-1 can promote mitosis in certain types of cells, such as mammalian vascular cells and hepatic stellate cells, and can also restrict the growth of specific cells, including neuroblastoma and bone marrow stromal cells. In some reports, Gal-1 regulates cell growth and proliferation in a dose-dependent, bidirectional manner. At low doses, Gal-1 has mitogenic effects and is affected by lactose. High dose Gal-1 inhibits cell proliferation, and this effect is independent of Gal-1's sugar binding activity [47]. In addition, Gal-1 also influences cell cycle progression in human breast cancer. Elevated levels of Gal-1 can enhance the proliferation of human thyroid cancer and glioma cells [160, 161]. Gal-1's regulation of cell proliferation might appear contradictory; however, it likely varies due to differences in cell type and condition, along with its intracellular and extracellular roles and polymeric state.

Gal-3 contributes to cancer cell proliferation by stimulating the growth of liver cancer, glioma, and pancreatic cancer cells [162]. Gal-4 is distinctly present in normal intestinal epithelial cells. and significantly reduced in colorectal cancer (CRC) samples. The elimination of Gal-4 expression can promote CRC cell proliferation and participate in cell cycle arrest [68].

Gal-7 has an inhibitory effect on cell proliferation, and knocking down Gal-7 can lead to excessive proliferation of human keratinocytes [76]. Gal-7 also has an inhibitory effect on cancer cell proliferation. Exogenous Gal-7 can inhibit the proliferation of human CRC cells and neuroblastoma [163, 164]. Further clarification is needed to understand the molecular mechanism through which Gal-7 regulates cell proliferation. It may be an effector of p53, and in the absence of Gal-7 or wild-type p53, it can lead to enhanced cell proliferation ability induced by UVB irradiation [165].

Gal-12 is involved in regulating the cell cycle, and its expression level increases in Jurkat T synchronized with G1 phase. Increased expression in the human cervical HeLa cell line also results in G1 phase cell cycle arrest [39]. Gal-12 is also an important intracellular regulatory factor in cortical cells, participating in the regulation of sebum cell cycle progression and proliferation [40].

**Fig. 3:**
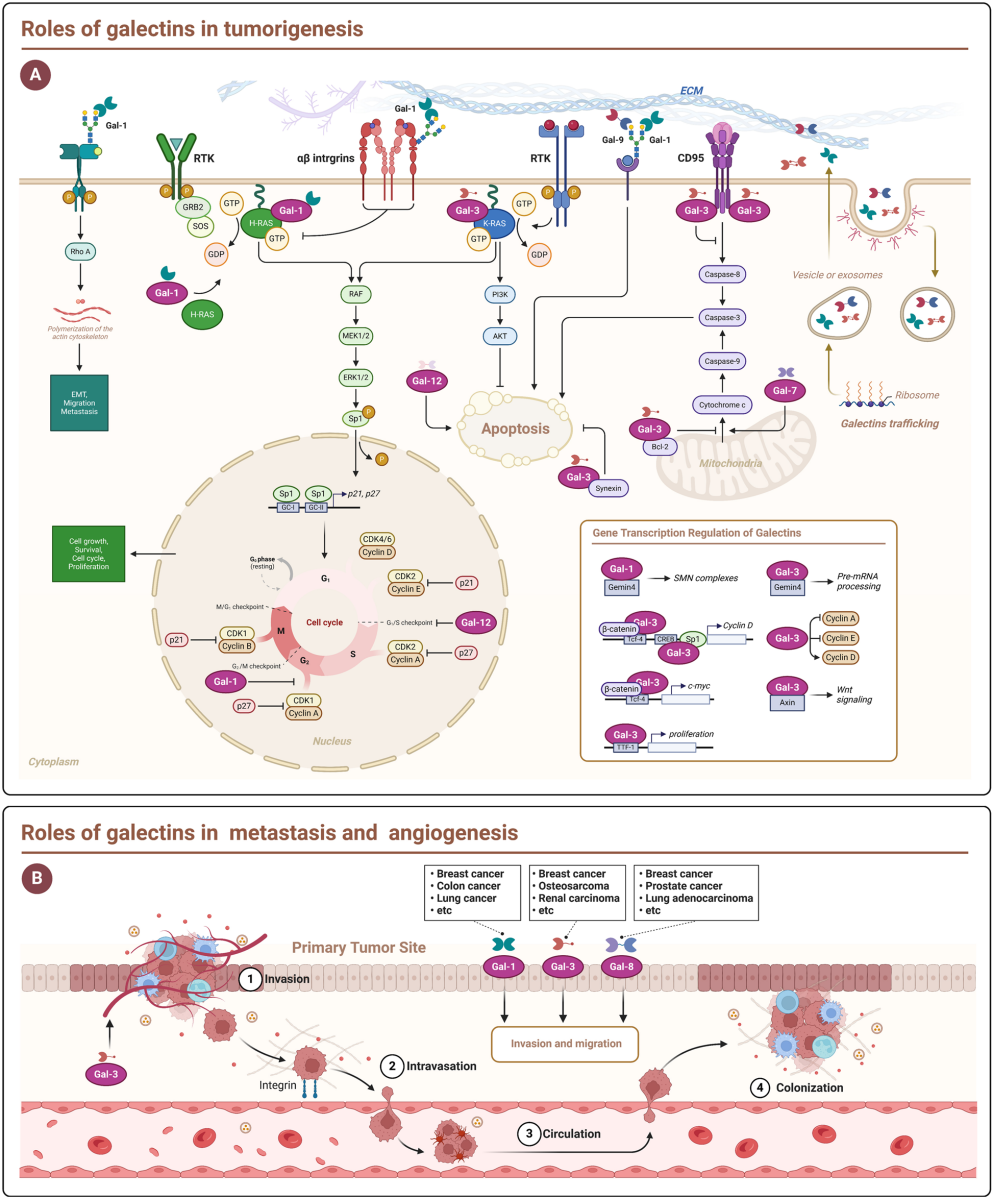
Biological functions of Galectins in tumor progression. **a** Roles of Galectins in tumorigenesis: Galectin is synthesized in the cytoplasm and secreted into the extracellular space through non-classical secretion pathways via vesicles or exosomes. It also functions intracellularly, performing various roles within the cell. Extracellularly, galectins can crosslink cell surface glycoconjugates modified with lactose-containing oligosaccharides to transmit signals into the cell. This mechanism regulates cell apoptosis, proliferation, transformation, and cycle progression. Intracellularly, galectin shuttles between the nucleus and cytoplasm, participating in cell growth, cell cycle progression, and apoptosis through intracellular signal regulation. Gal-1 and Gal-3 regulate the Ras/RAF1/ERK1/2 or PI3K/AKT pathways and mediate tumor transformation by interacting with oncogenes such as HRAS and KRAS. The interaction between extracellular dimer Gal-1 and α5β1 integrin inhibits the Ras/MEK/ERK pathway and continuously transcribes p21 and p27. The accumulation of p27 and p21 mediated by Gal-1 inhibits Cdk2 activity, leading to cell cycle arrest and growth inhibition. Gal-3 also regulates the levels of cell cycle inhibitors p21 and p27, as well as cell cycle proteins A, E, and D, resulting in cell cycle arrest. Although the molecular mechanism of Gal-12 in cell cycle arrest is not yet elucidated, it is involved in this process. Gal-1 interacts with Gemin4 and participates in the splicing pathway alongside the nuclear SMN complex. The increase in cell motility induced by Gal-1 involves an increase in RhoA expression and changes in actin cytoskeleton polymerization. Exogenous addition of Gal-1 and Gal-9 induces apoptosis in tumor cells, while Gal-7 and Gal-12 promote apoptosis through intracellular mechanisms. Gal-3 in the cytoplasm has an anti-apoptotic function by interacting with intracellular apoptosis regulators such as BCL-2. Gal-3 also interacts with various transcription regulatory factors in the nucleus, regulating gene expression and intervening in cell growth and mRNA splicing. **b** Roles of Galectins in metastasis and angiogenesis: The progression from a primary tumor to a metastatic tumor is a complex, multi-gene, multi-step process involving cell–cell and ECM adhesion, cell invasion and migration, and angiogenesis. Various galectins play crucial roles in different stages of this process. Gal-1, Gal-3, and Gal-8 influence tumor cell migration and invasion by binding to integrins or other cell surface proteins involved in cell migration and survival, as well as in angiogenesis. Additionally, Gal-3 can remodel cytoskeletal elements, particularly microfilaments, related to cell propagation, though the exact mechanisms remain undetermined. This remodeling affects the intrinsic movement of cells. Furthermore, Gal-3 promotes angiogenesis by facilitating the migration of endothelial cells. ECM: Extracellular matrix

The reports on the functionality of Gal-14 have mainly focused on regulating the immune response during pregnancy, with fewer reports on its impact on various types of tumors. Gal-14 seems to be able to promote tumor proliferation. Recent studies have shown that Gal-14 can promote HCC cell proliferation by enhancing heparan sulfate proteoglycan modification (Fig. 3) [166].

### Cell differentiation

Gal-7 is a marker of stratified epithelium and may have a potential connection with epidermal differentiation. Gal-7 can affect the differentiation of keratinocytes by regulating the JNK-miR-203-p63 pathway [76]. While Gal-7 exhibits a broad spectrum of biological effects, its role in tumor development remains incompletely understood. Several studies have indicated that the downregulation of Gal-7 expression may be related to poor differentiation of bladder squamous cell carcinoma and vulvar squamous cell carcinoma [72, 167]. Persistent poor differentiation of tumors is also a major feature of most malignant tumor cells.

The research reports on Gal-9's regulation of cell differentiation mainly focus on the differentiation and maturation of immune cells. Gal-9 induces the maturation of human DC and promotes Th1 cell-mediated immune response [168]. In vitro, Galectin-9 suppresses the differentiation of naïve T cells into Th17 cells. In combination with TGF-β, it enhances the expression of Foxp3 + regulatory T cells (Tregs), leading to apoptosis in Tim-3 + Th1 and Th17 cells [169–171]. Additionally, Galectin-9 participates in regulating the differentiation of mesenchymal stem cells into chondrocytes and the epithelial-mesenchymal transition (EMT) in Madin–Darby canine kidney epithelial cells (MDCK) cells. Moreover, it is involved in chondrocyte proliferation and differentiation [95].

Gal-12 primarily influences adipocyte differentiation, and its inhibition can impede the differentiation of 3T3-L1 cells prompted by adipogenic hormones [172]. Gal-12 is also expressed in macrophages. Reducing Gal-12 does not influence the differentiation of bone marrow cells into macrophages. However, it shifts macrophages towards the M2 type, increasing M2 marker levels and decreasing M1 pro-inflammatory factor expression [173, 174]. Gal-12 is specifically overexpressed in M3 type acute myeloid leukemia (AML), which is also referred to as acute promyelocytic leukemia (APL). Gal-12 helps to differentiate the tissue of APL cells, and inhibiting Gal-12 can promote granulocyte differentiation of APL cells, which might serve as a potential target for treating resistance in APL (Fig. 3) [175].

### Apoptosis

Various monosaccharide types combine with distinct components to create structurally diverse polysaccharides, which combine with proteins to produce various polysaccharide complexes. Cell surface apoptosis related receptor proteins can be modified by polysaccharides, leading to changes in protein conformation and function, and regulating signal transduction. Glycation does not depend on DNA as a template for regulation and is regulated by multiple repetitive factors. The exponentially differentiated polysaccharide structure and glycome lead to diverse proteomic regulation [176]. In the extrinsic apoptotic pathway, the glycosylation status of key initiator molecules, such as Fas ligand and TRAIL receptors, can influence receptor-ligand affinity, thereby affecting the activation of apoptosis [177–179]. Glycan-binding lectins serve as glycan ligands and are crucial regulators of apoptosis. The interaction between lectins and glycans induces glycosylated death receptor interactions. Galectins can modulate apoptosis signaling pathways by regulating the affinity between ligands and receptors, thus controlling tumor cell growth and various types of cell death [180].

The main biological function of galactose lectin includes its regulatory effect on cell apoptosis. Certain galectins trigger cell apoptosis by attaching to cell surface glycoproteins, whereas others modulate apoptotic signals via intracellular functions and protein interactions. Some of the pathways regulating apoptosis may be shared by various galectins [53].

Gal-1 induces apoptosis in activated T cells by activating Caspase-related pathways and promoting TCR chain phosphorylation. Gal-3 has anti-apoptotic activity, which is regulated by non-carbohydrate recognition. Gal-3's N-terminal and C-terminal sections are similar to Bcl-2, and there is a functional anti-death motif (NWGR) in the CRD region. It can bind to Bcl-2 in various types of tumor cells and inhibit mitochondrial apoptosis. Gal-3 binds to CD95/Fos and inhibits the activation of Caspase-8 by CD95/Fas [181]. Ca2 + and phospholipid binding protein Synexin can directly bind to Gal-3 and regulate its anti-apoptotic activity [115]. Gal-3 can interact with K-Ras to activate the PI3K/Akt pathway, thereby regulating cell growth and apoptosis [182]. Wild-type p53 can bind to the second intron region of the *lgals3* gene, inhibiting Gal-3 expression and inactivating its anti-apoptotic function [183]. Gal-4 is also an important immunomodulator, highly expressed and secreted into the tumor stroma in pancreatic ductal adenocarcinoma (PDAC), where it induces apoptosis in infiltrating T cells [184]. In intestinal epithelial cells, Gal-4 is selectively expressed and secreted. It binds efficiently to the CD3 epitope on activated peripheral and mucosal lamina propria T cells, inhibiting their activation while inducing apoptosis and cell cycle arrest [185].

Gal-7 and Gal-9 also tend to promote apoptosis. Gal-7 can activate the release of cytochrome c, initiating apoptosis [186]. It can induce apoptosis in Jurkat T cells, and this apoptotic induction is dependent on its lectin activity [187]. Studies on different tumor cell lines have confirmed that Gal-7 can make HeLa cells, DLD-1 colorectal cancer cells, DU-145 prostate cancer cells, and sarcoma-derived cell lines ST88-14 more sensitive to apoptotic stimuli. Downregulation of Gal-7 in cervical squamous carcinoma cells can increase resistance to chemotherapy-induced apoptosis [188–190]. These findings suggest that Gal-7 generally promotes apoptosis in these cell types. However, in some studies on breast cancer and melanoma, Gal-7 has shown a degree of anti-apoptotic activity [191].

Human and mouse recombinant Gal-9 treatment of thymocytes and immune cells can induce apoptosis in eosinophils, T lymphocytes, B lymphocytes, and macrophages [27]. Naive T cells develop into T helper type 1 (Th1) cells, expressing the Gal-9 receptor Tim-3. The interaction between the CDR region of Gal-9 and the β-galactoside domain of Tim-3 modulates intracellular calcium flux and cell aggregation capacity, leading to Th1 cell apoptosis and inhibition of Th1 cell-mediated immune responses [138]. Additionally, Gal-9 induces B cell apoptosis and mediates apoptosis in CD4 + and CD8 + T cells via the calcium-calpain-caspase-1 pathway [92]. Gal-9 is also capable of interacting with CD40 independently of Tim-3, thereby inhibiting the survival of CD4loCD40 + effector T cells [140].

Numerous studies have examined Gal-9's capability to trigger apoptosis in malignant tumor cells, both in vitro and in vivo. Gal-9 can directly activate apoptosis in hematologic malignancies, malignant melanoma, hepatocellular carcinoma (HCC), cholangiocarcinoma, and esophageal cancer cells in vitro [192–196]. The mechanisms of cell death vary among cell lines but are all directly activated by Gal-9, independent of tumor immunity activation. In multiple myeloma cells, Gal-9 activates the JNK/p38 pathway, activating caspase-3/8/9 to initiate apoptosis [197]. In chronic myeloid leukemia (CML), Gal-9-induced apoptosis depends on the intrinsic apoptotic pathway, activating ATF3 expression [192]. The receptor for Gal-9 in CML and myeloma remains unclear, but it likely depends on Gal-9’s carbohydrate recognition function, as lactose can antagonize Gal-9-induced apoptosis. The receptors for Gal-9 on the surfaces of HCC, cholangiocarcinoma, and gallbladder cancer cells are also unclear. No evidence of Tim-3 was found on the surface of HCC cells, suggesting the presence of other β-galactoside-glycosylated receptors [193].

Gal-12 exhibits anti-proliferative and pro-apoptotic functions, with its expression levels significantly downregulated in AML compared to normal controls (Fig. 3) [198].

### Tumor metastasis

The ability of tumors to invade and metastasize is a hallmark of malignancy. The metastatic process consists of several intricate steps, such as invading nearby tissues, entering blood or lymphatic vessels (intravasation), disseminating, exiting the vessels (extravasation), and colonizing distant secondary sites. A crucial phase in metastasis is the engagement between circulating tumor cells (CTCs) and the endothelial cells within secondary organs. This stage includes complex interactions between cell surface adhesion molecules such as integrins, ICAM, VCAM, and selectins [199].

Galectins play an undeniable role in the interactions between cells and the ECM, making their involvement in tumor metastasis significant (Fig. 3). The surface molecules of CTCs often undergo complex glycosylation modifications. Gal-1 present on CTC surfaces can attach to molecules like CD44, CD326, P-selectin, and membrane glycoprotein complexes (GPIIb/IIIa), facilitating tumor cell clustering, attachment to the ECM and endothelial cells, and retention in the capillaries of secondary organs, ultimately leading to the formation of metastatic lesions [8]. Research indicates that reducing Gal-1 expression in mouse breast cancer, colon cancer and other tumor cells can significantly reduce their lung metastasis ability [200]. In addition, this intervention method can to some extent alleviate the immunosuppressive state of tumors and improve the functional level of peripheral T cells [201].

Gal-3 is essential in many stages of tumor metastasis and can be actively or passively secreted by cells into the tumor microenvironment. It forms an oligomeric lattice with ligand molecules and sends signals to promote tumor migration and angiogenesis, mediating the process of ectopic adhesion between cells and ECM and regulating tumor cell detachment and metastasis from the primary site. Research has shown that Gal-3 binds to glycoconjugates in the ECM such as laminin, elastin, and type IV collagen [202, 203], and also interacts with the cell adhesion molecule integrin, activating regulatory factors such as FAK and Rac1, affecting the actin cytoskeleton and cell migration [204]. The dimerization and oligomerization of Gal-3 are associated with the clustering of glycosylated molecules during tumor metastasis. Elevated levels of Gal-3 have been observed in patients with metastatic renal cancer, where its overexpression enhances tumor cell motility and migration [205]. Silencing Gal-3 has been demonstrated to reduce the invasion and spread of multiple tumors, such as osteosarcoma and breast cancer [206].

Gal-4 acts as an intracellular tumor suppressor, and its overexpression can significantly reduce cancer cell migration and invasion [207]. In early-stage pancreatic cancer, Gal-4 is upregulated on the cell surface, functioning as an adhesion molecule. However, in the later stages of tumor progression, Gal-4 is downregulated, leading to the loss of cell–cell interactions, enhanced migratory ability, and tumor escape [208]. Contrarily, in some malignancies like poorly differentiated gastric cancer, inhibition of Gal-4 expression has been reported to reduce cancer cell proliferation and peritoneal metastasis [128].

Gal-7, acting as a p53 effector, is believed to support the removal of tumor cells and participates in the regulation of cell differentiation and apoptosis. However, Gal-7's complex role may also include promoting tumor progression. Some research indicates that Gal-7 regulates genes associated with metastasis, like MMP9, thereby enhancing the invasive potential of lymphoma and oral squamous cell carcinoma (OSCC) cells [209, 210]. Conversely, Gal-7 has been demonstrated to reduce the mobility of prostate cancer cells, thereby reducing their invasive potential [190].

Gal-8 enhances the expression of cytokines and chemokines, which are crucial for tumor progression in vivo. In Gal-8 knockout mice, breast cancer tumorigenesis was notably decreased, and lung cancer metastatic foci were considerably smaller. This suggests that cytokine and chemokine expression induced by Gal-8 aids in tumor development and metastasis [211]. Gal-8 is a cell matrix protein that regulates cell adhesion, promotes cell adhesion, diffusion and migration, and regulates the interaction between cells and matrix in various physiological and pathological processes [212, 213]. Cell adhesion to Gal-8 initiates an integrin-mediated signaling cascade. The role of Gal-8 enhances tumor cell aggregation and actively contributes to tumor metastasis [214].

Gal-9 shows anti-metastatic effects in cancer by preventing tumor cells from adhering to the extracellular matrix (ECM). This is achieved through the downregulation of ECM components, thereby blocking tumor invasion and metastasis [215]. In HCC, Gal-9 has been demonstrated to reduce cell migration and invasion [89]. Additionally, Gal-9 disrupts the binding of CD44 to hyaluronic acid, Very Late Antigen-4 (VLA-4), and Vascular Cell Adhesion Molecule-1 (VCAM-1), interfering with key steps in melanoma cell metastasis [216].

The migration of tumor cells mediated by galectins may be closely related to their interactions with integrins. For example, both Gal-1 and Gal-8 can bind to integrin β1, promoting cell adhesion and migration [217, 218]. In human breast cancer cells, high level of Gal-3 is associated with increased expression of integrin α6β1 and enhanced cell invasiveness [219]. Similarly, upregulation of Gal-7 in HeLa cells increases the expression of integrin α1, thereby regulating cell migratory ability [186]. Gal-9 facilitates prolonged cell adhesion to the basement membrane by mediating interactions between integrins and collagen-I as well as laminin-III.

Gal-14 is exclusively expressed in placental trophoblasts, where it facilitates their migration and invasion by increasing Akt phosphorylation and elevating the expression of matrix metalloproteinase MMP-9 and N-cadherin. This regulatory mechanism is involved in the development of preeclampsia and contributes to early pregnancy loss [220].

Grasping the factors and mechanisms that drive malignant tumor cell proliferation and metastasis is essential for enhancing tumor prognosis. This understanding is crucial for early cancer diagnosis and treatment, offering valuable insights into potential therapeutic targets.

### Angiogenesis

During tumor progression, factors such as vascular endothelial growth factor (VEGF) are secreted to promote the formation of new blood vessels, supplying solid tumors with necessary nutrients and oxygen. However, the vascular system in tumors often exhibits immature structures, leading to abnormalities in blood flow, vascular permeability, and lymphatic function. These irregularities can hinder immune cell infiltration. Suppressing tumor growth and boosting the immune system's capacity to target and destroy tumor cells can be achieved by inhibiting tumor angiogenesis and normalizing the structure and arrangement of tumor blood vessels (Fig. 3).

Gal-1 is abundantly present in the tumor microenvironment [221]. By activating signaling pathways like Raf/MAPK/MEK/ERK, it promotes endothelial cell proliferation and migration, thus boosting angiogenesis [222]. Additionally, Gal-1 regulates the expression of angiogenesis-related genes [223]. The ECM of tumor cells, rich in laminin and fibronectin, serves as key binding targets for Gal-1. Extracellular Gal-1 enhances physical interactions between cells, endothelial cells, and the ECM, supporting new blood vessel formation through its homotypic cross-linking abilities [224]. Numerous studies have reported that various O- or N-glycosylated targets of Gal-1 facilitate tumor angiogenesis [225].

Gal-3 is also crucial for tumor angiogenesis. It participates in VEGF and bFGF-mediated angiogenesis and can bind to VEGFR2, enhancing the response of VEGF-A in promoting angiogenesis [226]. Elevated levels of of Gal-3 in tumor cells induces macrophage infiltration and accelerates angiogenesis [227]. Gal-8, a crucial ECM molecule, plays a vital role in angiogenesis and is expressed in both normal and tumor-associated endothelial cells, as well as in lymphatic endothelial cells [228].

Conversely, Gal-7 and Gal-9 are among the few galectins that exhibit anti-angiogenic properties. Gal-7 can inhibit angiogenesis in vitro and in vivo, reducing blood vessel formation in colon cancer cell lines like DLD-1, effectively curbing tumor growth [164]. The pro-angiogenic activities of Gal-1, −3, and −8 are primarily related to VEGF, integrin signaling pathways, and the Ras signaling axis [229–231]. In contrast, the relationship between Gal-9 and these receptor signals remains unclear. Gal-9 primarily interacts with receptors like TIM-3, protein disulfide isomerase (PDI), CD40, and CD44, which play a greater role in the regulation of immune cell function and activity [232].

### Tumor immune microenvironment

With a deeper understanding of tumorigenesis and progression, recent research has increasingly focused on the tumor microenvironment. The glycan-binding abilities of galectin family members play crucial roles in tumor development. They not only act as intracellular signaling molecules regulating complex pathways that affect tumor cell apoptosis, metastasis, and angiogenesis but also serve as key participants in modulating signal crosstalk within the tumor stroma [49].

Most galectins can modulate the body's inflammatory response, aiding in tumor immune evasion. A key role of Galectin-1 in tumor development is converting the tumor microenvironment into an immune "cold" state, thus fostering an immunosuppressive environment that promotes tumor formation and growth. Tumor cells and various host cells show varying levels of Galectin-1 expression. Host-derived Galectin-1 mainly supports immune privilege for tumor cells [201]. Additionally, hypoxia-inducible factor (HIF-1α) regulates the expression of Galectin-1 [8]. The hypoxic tumor microenvironment further elevates Galectin-1 expression, exacerbating the immunosuppressive milieu within tumors.

#### T lymphocytes

During immune surveillance of tumor development, various immune cells, such as T lymphocytes, natural killer (NK) cells, and DCs, exhibit anti-tumor activities to varying degrees. However, T lymphocytes play the primary role in tumor immune defense. Galectins can influence the activity of effector NK cells and T cells. Research has indicated that surface glycoproteins on activated T cells, such as TCR, CD2, CD3, CD7, CD29, CD43, CD45, and CD95, act as primary receptors for Gal-1 [8, 233, 234].

**Fig. 4:**
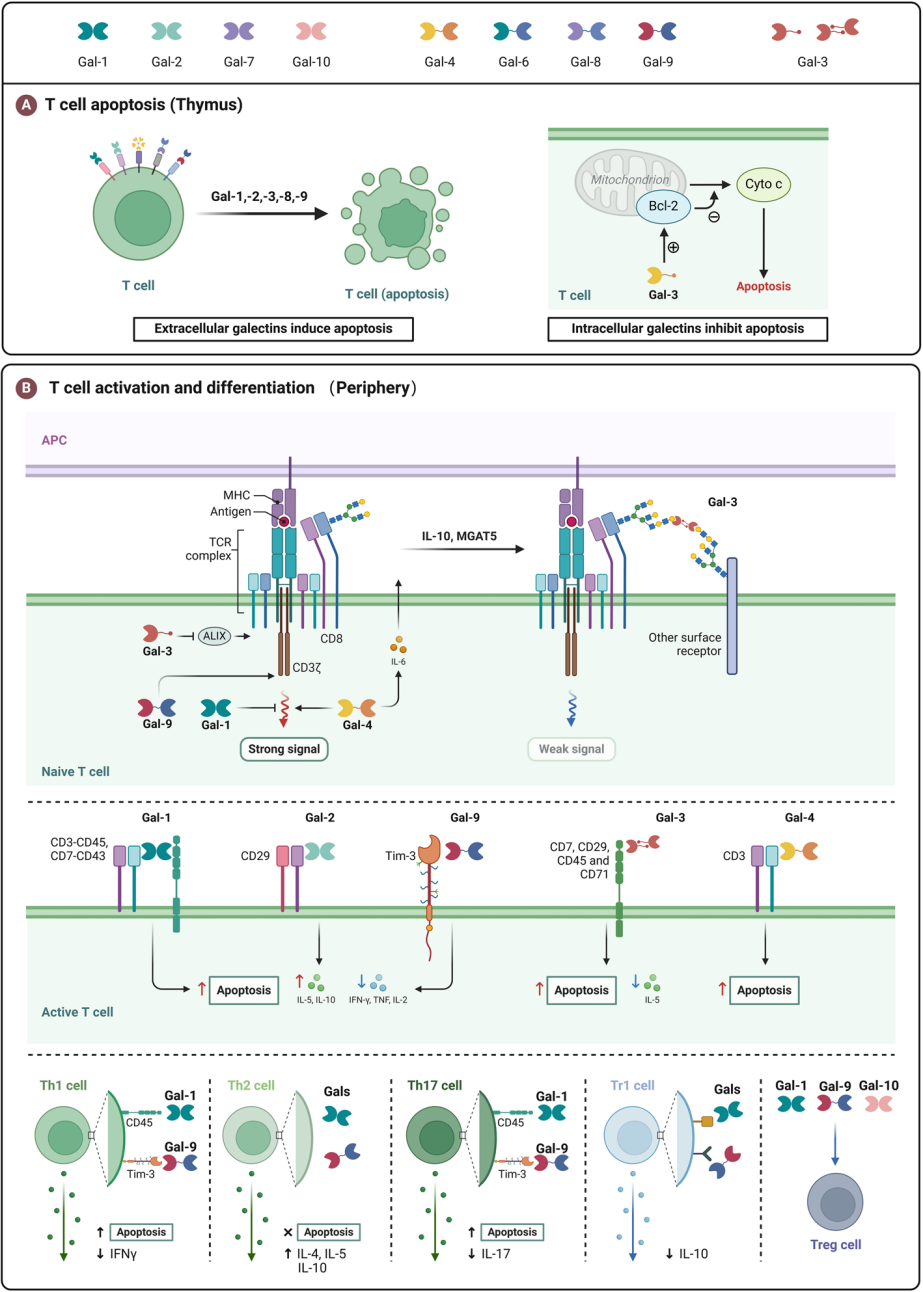
Roles of Galectins in T cell development. **a** T cell apoptosis (Thymus): In the thymic microenvironment, Gal-1, 3, 8, and 9 induce apoptosis in double-negative (CD4 − CD8 −) or double-positive (CD4 + CD8 +) thymocytes, suggesting their potential role in regulating central tolerance. Gal-1, 2, 3, 8, and 9 have been shown to induce T cell apoptosis in vitro. Additionally, Gal-3 can inhibit T cell apoptosis by interacting with the anti-apoptotic protein BCL-2. **b** T cell activation and differentiation (Periphery)**:** Gal-1 blocks early TCR-mediated activation signals, prolonging the survival of naive T cells, while Gal-4 triggers T cell activation and IL-6 production. Gal-3 forms lattices with complex N-glycans, limiting TCR clustering and increasing the agonist threshold for TCR signaling. Upon T cell activation, galectins 1, 2, 3, 4, and 9 bind to specific glycosylated receptors, including CD3, CD7, CD29, CD43, CD45, CD71, and Tim-3. This binding triggers various intracellular events that induce T cell death. Differential expression of cell surface glycoproteins or glycan structures leads to varying sensitivities of Th1, Th2, and Th17 cells to Gal-1 and Gal-9-mediated apoptosis. Galectins regulate the secretion of pro-inflammatory or anti-inflammatory cytokines and promote the generation of IL-10-producing Tr1 cells. Additionally, Gal-1 and Gal-10 contribute to the suppressive activity of CD4 + CD25 + Treg cells. TCR: T cell receptor; IL-6: Interleukin-6; Tim-3: T cell immunoglobulin and mucin domain 3; Th1: T helper 1; Tr1: T regulatory type 1 (Tr1) cell; Treg: Regulatory T cell

Gal-1 binds to N- and O-glycosylated T cell surface markers, activating intracellular signaling pathways that lead to apoptosis in activated T cells. This process involves multiple signaling axes, including the AP-1/Bcl-2-mediated apoptotic pathway, TCRζ chain phosphorylation-induced apoptosis, and the CD95/Caspase-8 death signaling pathway [180, 235, 236]. Other galectins, such as Gal-2, also exhibit apoptotic induction capabilities but primarily through binding to β-integrin rather than T cell markers like CD3 and CD7. Gal-2-induced apoptosis mainly activates Caspase-3 and Caspase-9, disrupts mitochondrial membrane potential, indicating a different apoptotic signaling pathway from Gal-1 (Fig. 4A) [110].

Gal-1-induced T cell apoptosis is selective and relies on the glycosylation status of T cell surface molecules. Glycosyltransferases and glycosidases influence glycan expression patterns and the glycosylation of T cell surface molecules (e.g., the N-glycans on CD45), creating glycosylation states that either promote or inhibit Gal-1 binding, thereby regulating T cell apoptosis and growth activation levels [234, 237–239]. Furthermore, research has demonstrated that Gal-1 greatly enhances the differentiation of Treg cells (CD4 + CD25 + FoxP3 +) and boosts their suppressive function [240]. Activation of Treg cells can also upregulate the synthesis and secretion of Gal-1 [241].

CTLA-4, a receptor that inhibits T cells, possesses two N-glycans. By binding to these N-glycans, Gal-1 enhances CTLA-4 retention on effector T cell surfaces, thereby strengthening growth arrest signals in T cells [234]. Th1 and Th2 cells also express galectin-binding receptors like CD7, CD43, and CD45, but Th2 cells exhibit resistance to Gal-1 binding due to sialylation of their N-glycans [242]. When Gal-2 is present, activated T cells tend to shift towards a Th2 profile [243]. Th1 and Th17 cells are susceptible to apoptosis induced by Gal-1 and can be selectively eliminated by Gal-1 [242]. Gal-1 stimulates T cells to increase the production of Th2 cytokines, such as IL-4, IL-5, IL-10, and IL-13, which may help mitigate autoimmune diseases during in vivo treatments [240, 242, 244]. Effector memory T cells (CD8 + CD45RO + CCR7 −) express reduced O-glycan structures, allowing them to evade Gal-1-induced apoptosis [245].

Gal-1 has been demonstrated to hinder T cell tumor-killing abilities by influencing their adhesion, migration, and recruitment functions. Gal-1 interferes with the actin cytoskeleton within T cells, reducing their adhesion and migration abilities, thereby preventing their movement to inflammation sites [246, 247]. At low physiological concentrations in early cancer stages, Gal-1 can promote the expression of endothelial Gal-9 and PD-L1, mediating tumor microenvironment rejection of T cells [248]. Additionally, Gal-1 alters cytokine secretion, increasing IL-10 and decreasing IFN-γ levels [249, 250]. Gal-1 interacts with numerous N- and O-glycosylated targets, triggering immunosuppressive signals and modulating the expression of cytokines related to the immune system. Though it is evident that Gal-1 significantly contributes to tumor immune evasion, the full scope of its interactions remains incompletely understood. Beyond its role in immune evasion, Gal-1 participates in numerous additional processes related to cancer.

Gal-1 plays a crucial role in regulating both adaptive and innate immune responses, impacting not only the tumor immune microenvironment but also various immune-related diseases (Fig. 4B). For example, Gal-1 can ameliorate chronic inflammation in autoimmune disease models [250]. In acute and allergic inflammation, Gal-1 signaling suppresses the movement of inflammatory cells [251]. Gal-1's capacity to inhibit T cell effector functions can enhance outcomes in organ transplants and graft-versus-host disease. Studies on pathogen infections (e.g., bacteria, viruses, parasites) have shown that Gal-1 can influence microbial activity and induce host cell apoptosis to control intracellular infections [252].

Gal-3 is another molecule involved in tumor immune evasion. It inhibits the apoptosis of activated T cells, blocks MHC-I molecule binding, and prevents NK cell activation [253]. Through interactions with CD7 and CD29, Gal-3 facilitates T cell apoptosis by promoting mitochondrial cytochrome c release and activating Caspase-3 (Fig. 4B) [116]. The interaction with Gal-3 and T cell surface glycoproteins CD45 and CD71 can induce T cell death [117]. Gal-4 directly impacts anti-tumor effector cells by engaging with the T cell receptor CD3, leading to T cell apoptosis. In PDAC tumor models, the knockout of Gal-4 substantially enhances infiltration of CD4 + and CD8 + T cells, alongside increased levels of activated CTLs, M1 macrophages, and antigen-presenting cells [184].

Gal-7, a potential effector molecule of p53, can promote the death of Jurkat T cells and human peripheral T cells when induced by p53 mutations, exhibiting immunosuppressive characteristics [187]. Gal-8 is a crucial regulator of immune responses, influencing both innate and adaptive immunity. It modulates the interactions between T cells and B cells in adaptive immune responses and regulates the activities of macrophages, DCs, and neutrophils in innate immunity [78]. High expression of Gal-8 in tumors and other immune disorders correlates with patient survival. Gal-8 induces tumor immune suppression by expanding regulatory immune cells (Tregs and MDSCs) and reducing CD8 + T cell infiltration [254].

Gal-9 acts as a diverse immune modulator, impairing cytotoxic lymphocytes and influencing various cell types within both innate and adaptive immunity [255]. Gal-9 functions as an eosinophil chemoattractant by recruiting these cells and facilitating superoxide production, while also attracting neutrophils and inducing DC maturation, triggering innate immune activation cascades [256, 257]. These mature DCs can migrate to nearby lymph nodes, induce T cell activation, and upregulate Th1 cytokines and costimulatory molecules (HLA-DR, CD40, CD54, CD80) [168].

**Fig. 5:**
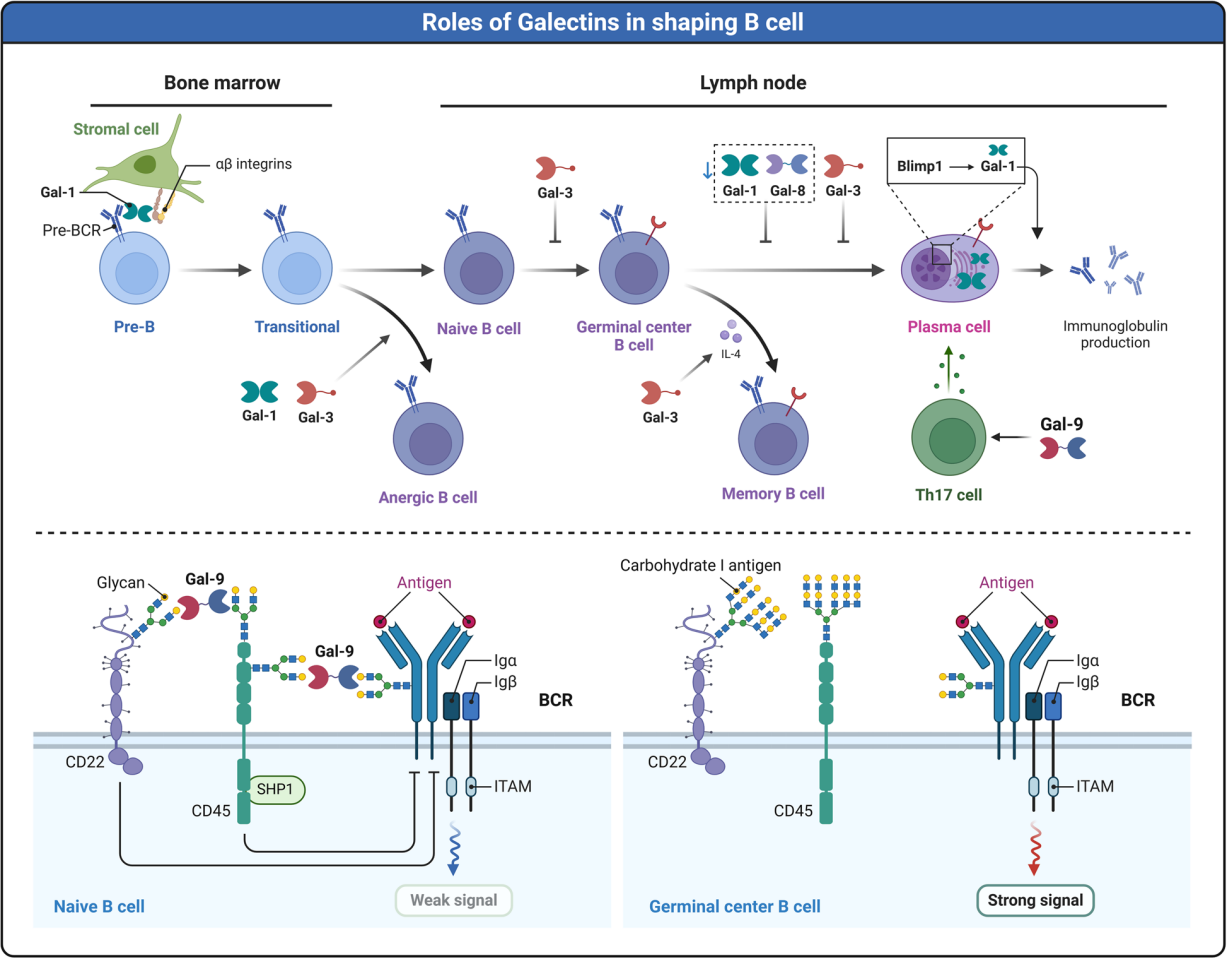
Roles of Galectins in shaping B cell. Galectins influence the differentiation, maturation, and signal transduction of B cells. During B cell development, Gal-1 secreted by stromal cells binds to glycosylated receptors such as α4β1 integrin, α5β1 integrin, α4β7 integrin, and the pre-BCR. When B cells enter the periphery, Gal-3 impairs their differentiation into plasma cells. Endogenous Gal-9 enhances the surface glycosylation of transmembrane proteins CD22, CD45, and BCR binding in immature B cells. CD45 mediates the recruitment of the phosphatase SHP1, inhibiting BCR signaling, suppressing B cell activation, and preventing differentiation into plasma cells. In the germinal center, B cells express carbohydrate I antigen, reducing Gal-9 mediated inhibitory signaling. Downregulation of Gal-1 and Gal-8 can impair B cell differentiation into plasma cells. Gal-3 can also hinder the development of germinal center B cells by inhibiting IFN-γ production and Tfh cell differentiation. However, Gal-3 supports B cell survival and promotes IL-4-induced differentiation into memory B cells. Mature B cells activate the transcription factor BLIMP1, upregulating Gal-1 expression, and promoting plasma cell differentiation and immunoglobulin production. Gal-9 appears to enhance plasma cell IgA production by promoting Th17 cell differentiation. In unconventional, incompetent B cells, the expression of Gal-1 and Gal-3 is upregulated, suggesting their involvement in regulating B cell tolerance. Pre-BCR: Pre B-cell receptor; BLIMP1: B lymphocyte-induced maturation protein 1

Gal-9 is essential for managing T cell development and maintaining homeostasis. It is highly expressed in thymic epithelial cells and participates in the selection of developing thymic T cells. High-concentration, short-term Gal-9 stimulation activates the calcium-calpain-caspase-1 pathway, mediating T cell apoptosis [258]. In contrast, prolonged exposure to low concentrations of Gal-9 activates resting memory T cells and encourages their differentiation into a Th1 phenotype [259]. Gal-9 facilitates naive T cells differentiating into Tregs by enhancing Foxp3 expression and suppressing the formation of Th17 cells [171]. Regardless of Tim-3 expression, Gal-9 does not influence Treg apoptosis. Tim-3, a recognized regulator of T cell immune responses and a prominent Gal-9 receptor, mediates Gal-9 signaling. For Tim-3-negative Tregs, Gal-9 can bind to its receptor PDI, promoting Treg migration. Placenta-specific galectins, Gal-13 and Gal-14, also influence immune responses by triggering apoptosis in activated T cells. They can migrate and kill maternal decidual T cells and macrophages, and regulate neutrophil activity in the placenta [260, 261]. Gal-13 and Gal-14 enhance CD95 expression on the surfaces of cells. Additionally, Gal-13 increases CD25 expression on T cells, while Gal-14 reduces CD71 expression. Unactivated T cells produce more IL-8 in the presence of these two galectins (Fig. 4B) [262]. While we have provided a comprehensive review of the role of galectins in T cell functions, the significance of galectins in B cell differentiation, maturation, and signal transduction is equally noteworthy, underscoring the pivotal role of galectins in the overall immune response (Fig. 5)

#### Other immune cells

**Fig. 6:**
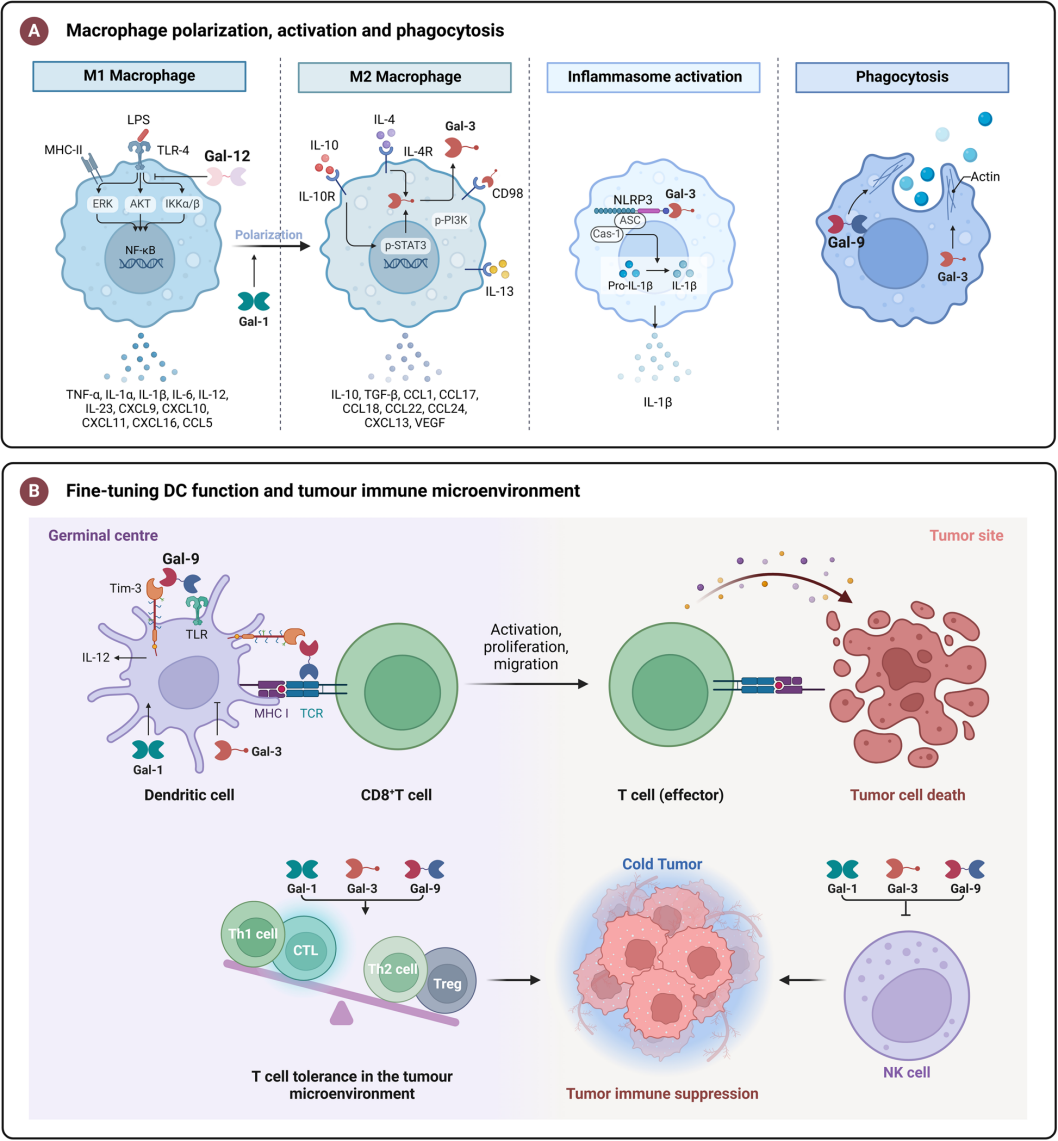
Roles of Galectins in regulating innate immunity and tumor microenvironment. **a** Macrophage polarization, activation and phagocytosis: Gal-12 is a positive regulator of LPS-induced polarization of M1 macrophages. It primarily achieves this by regulating downstream components such as IKKα/β, AKT, and ERK of TLR4, reducing NF-κB activation and ultimately leading to M1 polarization. Conversely, Gal-3 promotes M2 polarization of macrophages. IL-10 mediates the activation of the transcription factor STAT3, promoting Gal-3 expression. IL-4 can also induce Gal-3 expression, which promotes M2-like polarization of macrophages by binding to CD98. Additionally, Gal-3 interacts with the NLRP3 inflammasome, promoting the production of IL-1β. Both Gal-3 and Gal-9 regulate macrophage phagocytosis by controlling dynamic changes in cytoskeletal proteins. **b** Fine-tuning DC function and tumor immune microenvironment: Gal-9 supports DC production of IL-12 and synergizes with TLRs by connecting with Tim-3 to initiate adaptive immune responses. Gal-9 enhances tumor-specific T cell responses by promoting Tim-3-dependent DC-CD8 T cell interactions. Gal-1 promotes the maturation of DCs, enhancing their migration phenotype. DCs from Gal-3-deficient mice exhibit defective migration but secrete higher levels of IL-12 and have an increased potential for T cell stimulation. In the tumor microenvironment, galectins such as Gal-1, Gal-3, and Gal-9 target CTLs and Th1 cells, shifting the balance of T cell homeostasis towards Th2 and Treg cells. This shift promotes T cell tolerance in the tumor microenvironment, contributing to a "cold state" of tumor immunity. DC: Dendritic cell; TLR: Toll-like receptor; CTL: Cytotoxic T lymphocytes

Tumor-associated macrophages (TAMs), originating from monocytes, form a vital part of the tumor microenvironment and display the anti-tumor M1 type during early stages. As tumors progress, macrophages may shift to the pro-tumor M2 phenotype, aiding in creating a microenvironment that supports tumor growth, survival, and metastasis [263]. Numerous studies have demonstrated that Gal-1 can affect the transition of macrophages from the M1 phenotype to M2 (Fig. 6). Gal-1 decreases the expression of the M1 macrophage marker MHC-II [264]. Knocking down Gal-1 levels in the tumor microenvironment decreases M2 macrophage numbers and diminishes immunosuppressive cytokine expression [265, 266]. Additionally, beyond regulating macrophage polarization, Gal-1 can induce apoptosis in monocyte precursors to macrophages [267], even though it does not significantly induce apoptosis in mature macrophages. Overall, Gal-1 aids in creating an immunosuppressive microenvironment that supports tumor cells.

Within the tumor microenvironment, DCs display varied characteristics; they may activate T cells by providing signals or modulate immune responses through cytokine secretion. The regulation of DCs by Gal-1 is complex and appears contradictory (Fig. 6). Physiological levels of Gal-1 seem to promote tolerogenic DCs, while high concentrations of Gal-1 induce a pro-inflammatory response in DCs [268]. Research by Fulcher et al. reported that Gal-1 promotes the functional maturation and migration of monocyte-derived DCs. Conversely, Thiemann et al. discovered that Gal-1 suppresses the migration of immunogenic DCs [269]. Additionally, some studies indicate that Gal-1 impairs the differentiation of immature DCs, enhancing the tolerance of mature DCs [270]. Further research in neuroblastoma and lung cancer has revealed that tumor-derived Gal-1 inhibits DC maturation [271, 272].

Various molecules secreted within the tumor microenvironment can promote the downregulation and exhaustion of NK cells, weakening their cytotoxic capabilities. In glioma cells, overexpressing Gal-1 enables evasion of NK cell immune surveillance. Conversely, losing Gal-1 reinstates the cells' sensitivity to NK cell-mediated cytotoxicity [273]. Myeloid-derived suppressor cells (MDSCs), which have immunosuppressive functions, also show reduced infiltration when Gal-1 is absent in glioma cells [265, 274]. Overall, Gal-1 worsens tumor progression by hindering the cytotoxic activity of anti-tumor immune cells and facilitating the proliferation and infiltration of immunosuppressive cells.

In the tumor microenvironment, macrophages, DCs, and mesenchymal stromal cells (MSCs) can express Gal-3. Gal-3 has been found to inhibit NK cell surveillance, not by directly killing NK cells but by binding to regulatory molecules on tumor cell NK cell receptors [253]. MSCs produce various immunosuppressive factors in the tumor microenvironment and express high levels of Gal-3, though the specific role of MSC-derived Gal-3 remains unclear [275, 276].

For NK cells, Gal-9 impairs their cytotoxicity and cytokine production efficiency through Tim-3-independent pathways (Fig. 6) [277]. Moreover, Gal-9 facilitates the proliferation of immunosuppressive macrophages by activating monocyte AP-1 and NF-IL6, thereby inducing pro-inflammatory cytokine expression [278]. The impairment of NK cell function by Gal-9 likely contributes to the immunosuppressive state of the tumor microenvironment. Gal-9 downregulates several immune activation genes in NK cells, damaging lymphokine-activated killing and Tim-3-independent IFN-γ production pathways [277]. Research indicates that Gal-9 expression is increased in HCC cells when compared to normal liver cells [279]. Gal-9 aids in inducing stable aggregation of tumor cells, inhibiting their detachment and metastasis, thereby exerting an anti-proliferative effect [89]. In vitro, Gal-9 has the ability to induce apoptosis in HCC cells in a manner dependent on both dose and time [193].

In summary, Gal-9 likely exhibits a "double-edged sword" effect in tumors. It has the potential to prevent metastasis and induce apoptosis, but it also promotes the establishment of a tolerant tumor immune environment. Therefore, Gal-9 might be upregulated in the early stages of tumor development to help establish immune tolerance and then be lost as the tumor progresses, reducing its anti-metastatic and pro-apoptotic capabilities.

### Others

Gal-1 exhibits diverse expression patterns in different types of tumor cells and ECM, and is involved in regulating tumor cell proliferation and death, invasion and metastasis, angiogenesis, and immune escape. In addition, Gal-1 also exhibits specific expression patterns in various non tumor tissues. During the initial phases of human embryonic development, Gal-1 expression is associated with connective tissue, muscle tissue, skin, gonads, thyroid, kidney, and other organs [47]. Gal-1 in the endometrium plays a role in regulating both the menstrual and pregnancy cycles [280]. Gal-1 is extensively distributed and significantly influences the development of neural networks within both the central and peripheral nervous systems of rodents [281]. In addition, Gal-1 is involved in muscle development and regeneration during myogenic differentiation and is regarded as a potential therapeutic tool for human muscle malnutrition [282]. Many studies suggest that Gal-1 might play a crucial role in the differentiation of hematopoietic cells. It is possibly involved in regulating the proliferation of hematopoietic stem and progenitor cells, as well as osteoblast differentiation [283]. Furthermore, Gal-1 is crucial in the development of primary sensory neurons and the formation of synaptic connections in the spinal cord during embryogenesis. Its expression level is linked to numerous neurological disorders. Oxidized Gal-1 can function as an autocrine or paracrine factor to enhance axonal regeneration [7].

Gal-10 forms hexagonal and bipyramidal Charcot-Leyden crystals (CLCs) in vivo, which serve as potential biomarkers for eosinophilic diseases and play a functional role in immunity [32]. CLCs are identified by the NLRP3 inflammasome, and once engulfed by macrophages, they stimulate the release of the pro-inflammatory cytokine IL-1β [284]. Additionally, CLCs recruit neutrophils to epithelial cells and secrete cytokines that activate neutrophil functions [285]. Some studies suggest that Gal-10 helps CD25 + Treg cells regain proliferative capacity and diminishes their immunosuppressive function [97]. However, the effect of Gal-10 on regulatory T cell function is debated, as other research indicates that Gal-10 does not significantly impact the viability of regulatory T lymphocytes or the expression of the transcription factor FOXP3.

## Role of galectins in malignancies

**Fig. 7:**
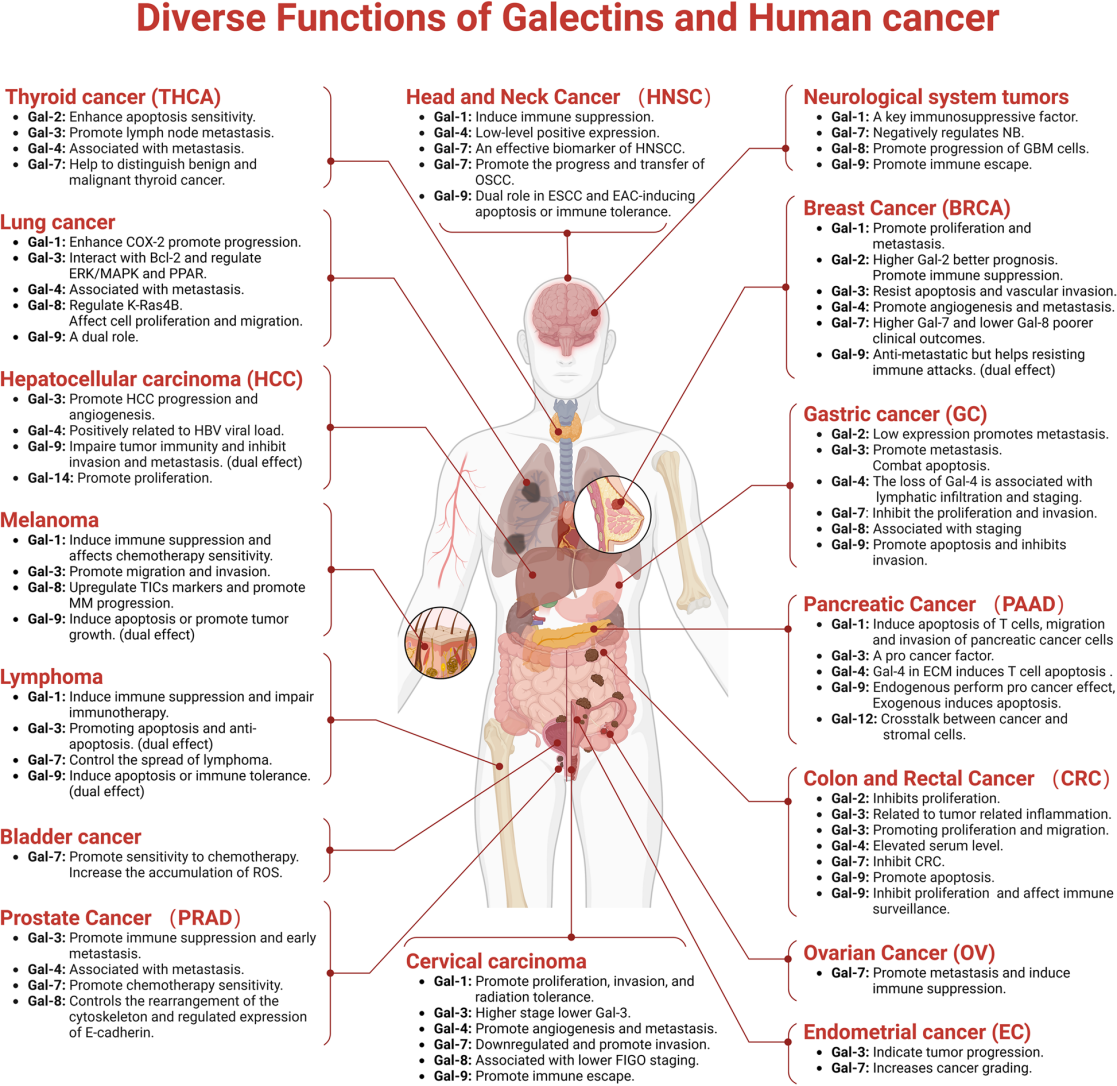
Diverse functions of galectins and human cancer

Alterations in the glycosylation patterns of cell membrane proteins and lipids influence cellular interactions, interactions with the ECM, and the properties of cell surface receptors. Galectins are involved in regulating tumor cell transformation, metastasis, angiogenesis, and immune evasion. Elucidating the key mechanisms involved will promote the application of these multifunctional targets in cancer therapy (Fig. 7). Gal-1, −3, −7, and −9 have all been reported in various types of tumors, but they play differing roles in cancer. In cancer patients, multiple family members of galectins are found to be elevated in circulation. Elevated serum levels of Gal-2, −3, −4, and −8 may be involved in promoting metastasis, showing adverse effects in colon cancer, breast cancer, lung cancer, and others [60].

Gal-1 has been the most extensively studied galectin and is secreted abundantly in almost all malignant tumor cells, impacting the biological phenotype of tumor cells. This includes colon cancer, melanoma, prostate cancer, ovarian cancer, bladder cancer, and involves tumor apoptosis, invasion, migration, cell cycle regulation, and immune regulation [286]. Gal-1 facilitates tumor cell proliferation and is involved in immune evasion by tumors, potentially serving as a crucial molecular target for cancer treatment.

Gal-3 typically promotes tumor progression and is linked to the development and malignancy of various cancers, including pancreatic cancer, hepatocellular carcinoma, renal cell carcinoma, esophageal squamous cell carcinoma, nasopharyngeal carcinoma, melanoma, glioblastoma, and lymphoma. Interestingly, in certain cancers like breast cancer, prostate cancer, endometrial cancer, and leukemia, Gal-3 can function as a tumor suppressor. The contribution of Gal-3 to tumors is not fully understood; different cancer types and subcellular localizations may lead to different impacts. Whether it can serve as a cancer diagnostic or therapeutic marker remains to be seen, but most researchers believe that studying Gal-3 can help improve cancer diagnosis and prognosis.

Different galectins have very different biological impacts in various types of cancer, and the variation in interacting partners across different cancers might be a key factor causing these differential effects.

### Lung cancer

Lung cancer is the most common malignancy in terms of incidence rates. High Gal-1 expression is linked to poor prognosis in lung cancer, as elevated levels are found in both the serum and tumor tissues of patients. In non-small cell lung cancer (NSCLC) cell lines, Gal-1 overexpression boosts COX-2 and its metabolite prostaglandin E2 (PGE2) expression, thereby facilitating tumor progression.

In lung cancer, Gal-3 promotes progression by interacting with the anti-apoptotic protein Bcl-2 and regulating the ERK/MAPK and PPAR signaling pathways. Gal-4 expression in lung cancer may be linked to tumor metastasis, with higher levels observed in metastatic tissue samples. Subcellular localization of Gal-4 in metastatic cells is enriched in the cytoplasm and nucleus [287]. Contrarily, other studies have shown that in the lung cancer cohort of smokers, the *LGALS4* gene is hypermethylated and downregulated, suggesting its function is suppressed [21]. Gal-8 interacts with K-Ras4B, influencing its farnesylation, which in turn modulates K-Ras effector pathways, as well as cell proliferation and migration. In lung and pancreatic cancer cells, siRNA-mediated suppression of Gal-8 raises K-Ras4B levels and enhances ERK1/2 activity, ultimately reducing cell migration and proliferation [135].

Gal-9 has a dual role in lung cancer. It is positively expressed in various pathological types of NSCLC. In NSCLC cells, elevated Gal-9 expression is linked to prolonged survival, while high levels of Gal-9 in tumor-infiltrating lymphocytes (TILs) are associated with early tumor recurrence. Research on small cell lung cancer (SCLC) revealed that reduced Gal-9 expression is linked to a higher immune risk score and a greater likelihood of early recurrence. Within the cytoplasm of tumor cells, Gal-9 enhances the adhesive properties of lung cancer cells, reducing their metastatic potential [288]. Treatment with recombinant human Gal-9 can induce macrophage differentiation into plasmacytoid DC-like macrophages (Mφs) in vivo, enhancing NK cell activation and prolonging the survival of mice with lung cancer [289]. The interaction between Tim-3 and Gal-9 contributes to both primary and secondary resistance to PD-1 therapy in lung cancer.

### Gynecological tumors

#### Breast cancer

Breast cancer is a common malignancy in women. Although improvements in screening and treatment have lowered mortality rates, breast cancer incidence keeps increasing every year. Therapies targeting progesterone receptors (PR), estrogen receptors (ER), and human epidermal growth factor receptor 2 (HER2) have significantly improved patient prognosis. However, addressing triple-negative breast cancer (TNBC) is still difficult due to the absence of these molecular targets [290, 291].

The function of galectins in breast cancer remains incompletely understood. Gal-1 expression is associated with the invasive and metastatic capabilities of breast cancer. Inhibiting Gal-1 expression can reduce breast cancer proliferation and lung metastasis. Additionally, tumor cell-secreted Gal-1 can increase CD4 + CD25 + Foxp3 + Treg cells, promoting an immunosuppressive microenvironment in breast cancer [292]. Gal-2 overexpression has been linked to a favorable prognosis in breast cancer [293]. A multi-dimensional CRISPR screen targeting disease-related immune genes identified Gal-2 as a candidate involved in TNBC immune evasion. Gal-2 promotes macrophage M2-like polarization and proliferation through the activation of the colony-stimulating factor 1 (CSF1)/CSF1 receptor (CSF1R) axis, thereby enhancing tumor growth in vivo but not in vitro. Blocking Gal-2 expression can reverse immunosuppression and inhibit tumor growth, making Gal-2 a potential target for TNBC immunotherapy [294]. Gal-3 levels are markedly higher in breast cancer tissues compared to nearby non-cancerous tissues, showing significant expression in TNBC and increased serum concentrations in breast cancer patients [65]. Some studies suggest that Gal-3 expression is associated with enhanced tumor angiogenesis, shorter disease-free survival, and lower overall survival rates [295]. Though Gal-3 expression is not yet confirmed as an independent prognostic marker for breast cancer, it is linked to chemotherapy resistance. In breast cancer, Gal-3 can bind to Bcl-2 to initiate anti-apoptotic functions and enhance metastatic potential, as well as promote cancer progression through binding and activating K-Ras.

In breast cancer patients, serum Gal-4 levels are elevated to 11 times those in healthy individuals, promoting angiogenesis and tumor metastasis [296]. Elevated Gal-4 and its ligand MUC1 in the serum of advanced breast cancer patients may serve as markers for evaluating post-treatment metastasis. Gal-7 expression increases in breast cancer, particularly in high-grade tumors, HER-2 overexpressing tumors, and basal-like subtypes, promoting metastatic behavior. This appears contradictory given that Gal-7 is a p53-induced gene. However, mutant p53 in breast cancer cells can also induce Gal-7 expression via NF-κB and transcription factor C/EBPβ−2 [22]. Poor clinical outcomes are linked to elevated Gal-7 expression and reduced Gal-8 expression. High levels of cytoplasmic Gal-7 are linked to HER2 and PR status, patient age, and tumor grade. Conversely, elevated Gal-8 expression is significantly associated with better overall survival (OS) (*P* = 0.032) [297]. In the breast tumor microenvironment, the transmembrane glycoprotein PDPN can bind to LEC-derived Gal-8, promoting integrin β1 activation in a glycosylation-dependent manner, leading to local matrix remodeling and increased angiogenesis and lymphatic invasion in breast cancer [134].

By enhancing tumor cell adhesion, Gal-9 possesses anti-metastatic potential in breast cancer. However, breast cancer cells can express LPHN1 and its ligand FLRT3, activating the Gal-9-Tim-3 pathway to protect against immune attacks [298].

#### Cervical cancer

Cervical cancer ranks as the second most prevalent gynecological malignancy globally, and it is strongly linked to high-risk HPV infection. However, many non-infected patients may experience disease progression due to genetic abnormalities and other factors. In cervical tissues, Gal-1 expression rises with pathological grade and is strongly linked to the depth of cancer invasion and lymphatic metastasis [299]. The primary functions of Gal-1 in cervical cancer cells are to promote proliferation and invasion. Downregulating Gal-1 expression significantly reduces the invasive capability of cervical cancer cells [300]. Gal-1 interacts with H-Ras to activate downstream signaling pathways and repair DNA damage caused by radiotherapy. As a hypoxia-responsive protein, Gal-1 contributes to the radioresistance of cervical cancer [301].

Gal-3 expression is downregulated in cervical cancer tissues, decreasing progressively from low-grade squamous intraepithelial lesions (LSIL) to high-grade squamous intraepithelial lesions (HSIL) and invasive squamous cell carcinoma (ISCC). Gal-7 is also expressed at low levels in cervical cancer tissue and can induce MMP-9 through the p38/MAPK signaling pathway, promoting cell invasion. Gal-9 expression is likely related to malignant transformation in cervical cancer, with significantly lower expression in HSIL compared to LSIL. In cervical squamous carcinoma and HSIL, Gal-7 expression is downregulated. Increased Gal-7 expression in cervical cancer is often associated with heightened sensitivity to concurrent chemoradiotherapy (CCRT), although Gal-7 alone is not an independent predictor of CCRT sensitivity. It can, however, be used in conjunction with other proteins like S100A9. In cervical squamous carcinoma, the expression levels of both Gal-7 and S100A9 are decreased, showing a negative correlation with cancer staging and lymph node metastasis. Knocking out Gal-7 and S100A9 increases MMP-9 expression and activates the PI3K/Akt signaling pathway, enhancing cancer cell proliferation [302].

The expression of Gal-8 and Gal-9 in 250 cancer tissue samples was evaluated using an immunoreactivity score (IRS). In squamous cell carcinoma (SCC) patients, Gal-8 is linked to earlier FIGO stages and has a positive correlation with recurrence-free survival. High levels of Gal-9 and Tim-3 expression are observed in cervical cancer. The Tim-3-Gal-9 pathway activation encourages Treg cells to release TGF-β and IL-10, which suppresses the cytotoxic activity of Th1 and CD8 T cells, thus facilitating immune evasion by cervical cancer cells. Additionally, the promoter regions of Gal-9 and Tim-3 in cervical cancer exhibit hypomethylation. Altering the methylation status of Gal-9 and Tim-3 can reverse their expression levels, improving the immune status of the tumor microenvironment [303].

#### Endometrial cancer

Research on Gal-3 in endometrial cancer is still limited, lacking large-scale clinical studies and long-term follow-ups. Further evidence is required to assess the direct link between Gal-3 and the progression and malignancy of endometrial cancer [304]. Some studies indicate that elevated Gal-3 expression correlates with the histological grade and tumor type, suggesting progression in endometrial cancer [305, 306]. In contrast, some studies have found that reduced Gal-3 expression in endometrial cancer inhibits tumor growth and is linked to a poorer prognosis in patients. As the grade of cancer increases, the production of Gal-7 also rises in endometrial cancer, promoting metastasis by reducing intercellular adhesion.

#### Ovarian cancer

Gal-7 is upregulated in epithelial ovarian cancer cells but undetectable in normal ovarian tissue. Its expression correlates positively with tumor grade, older age, and higher mortality rates in ovarian cancer. Gal-7 also induces MMP-9, promoting the metastasis of ovarian cancer cells. Similar to other galectins, Gal-7 can induce an immunosuppressive state in the tumor microenvironment by causing T cell apoptosis [307]. The expression variability of Gal-8 in ovarian cancer depends on the histological type, subtype, stage of progression, and degree of tumor differentiation. In high-grade serous carcinoma (HGSC), Gal-8 expression is related to chemotherapy resistance and decreased survival rates [308]. In ovarian cancer patients, Gal-9 plays a dual role in influencing overall survival (OS) and disease-free survival (DFS). Compared to Gal-9 negative cases, Gal-9 expression typically correlates with a poorer prognosis; yet, those with high Gal-9 levels may achieve the most favorable outcomes [309].

### Melanoma

Melanoma cells secrete Gal-1, which acts as a key immunosuppressive factor. Blocking Gal-1 in tumor tissues can inhibit tumor growth and enhance Th1-type anti-tumor immune responses [310]. Melanoma cell adhesion molecule (MCAM) binds to Gal-1 through its N-glycans and is one of the primary ligands for Gal-1. Reducing Gal-1 expression in melanoma cells can increase the chemotherapy sensitivity of tumor-bearing mice [311]. Gal-3 facilitates melanoma progression by boosting cell migration and invasion in vitro, as well as metastasis in vivo, via AP-1 transcription.

Hypoxia triggers glycan changes in tumor-initiating cells (TICs) and metastatic melanoma (MM) cells, such as the activation of Gal-8 and the reduction of the I-branching enzyme β1,6 N-acetylglucosaminyltransferase 2 (GCNT2). In MM patients, increased serum Gal-8 elevates NGFR/CD271 expression, a TIC marker, thereby enhancing TIC activity and contributing significantly to MM progression and treatment resistance. Silencing GCNT2 increases TIC marker levels and tumor initiation potential in vivo. Thus, elevated Gal-8 and reduced GCNT2 may serve as biomarkers for MM [312]. The reduction or absence of Gal-9 is closely related to melanoma metastasis and progression, and Gal-9 has a direct apoptosis-inducing effect on melanoma cells. In patients with metastatic melanoma, high serum levels of Gal-9 tend to support tumor growth. Gal-9 interacts with CD206 on M2 macrophages, promoting angiogenesis and the release of pro-tumor growth chemokines, which results in a poor prognosis [313]. In melanoma, the methylation levels of Gal-9 and Tim-3 are strongly linked to immune cell infiltration [314].

### Lymphoma

Lymphomas primarily include classical Hodgkin lymphoma (cHL) and non-Hodgkin lymphoma (NHL). In cHL, an abnormal type of B lymphocyte known as Reed-Sternberg (RS) cells is present. These RS cells use an AP1-dependent enhancer to overexpress Gal-1. Suppressing Gal-1 expression can enhance T cell activity and rebalance the Th1/Th2 ratio [240]. Diffuse large B-cell lymphoma (DLBCL) and primary mediastinal large B-cell lymphoma, among other Hodgkin lymphoma types, do not express Gal-1. In NHL, elevated Gal-1 expression counteracts CD20 immunotherapy in mice, and exogenous recombinant Gal-1 impairs macrophage activation and function [315]. Gal-3 is expressed in non-mediastinal DLBCL and primary effusion lymphoma (PEL) patients and cell lines but not in Burkitt lymphoma, low-grade follicular lymphoma (FL), marginal zone lymphoma, or small B-cell lymphoma. Gal-3 interacts with the cell surface protein CD45, reducing tyrosine phosphatase activity, which helps DLBCL cells resist apoptosis [316]. In adult T-cell leukemia/lymphoma (ATLL), stromal cells extensively express Gal-3, which induces apoptosis by binding to CD7. In patients with non-acute promyelocytic leukemia, serum Gal-3 levels are notably higher than in controls, and those expressing higher levels of Gal-3 experience a shorter overall survival time. Although the mechanisms need further investigation, Gal-3 can be used as a prognostic marker for poor outcomes in lymphoma. Gal-7 is constitutively expressed in lymphomas and can control the spread of lymphoma cells by regulating MMP-9 expression [317]. In lymphomas such as cutaneous T-cell lymphoma (CTCL), Gal-9 is highly expressed and reduces CD8 T cell infiltration. Exogenous recombinant Gal-9 can induce apoptosis in CTCL cells independently of Tim-3 activation [318]. In DLBCL patients, Gal-9 frequently exhibits mutations that interfere with its interaction with Tim-3 [319].

### Head and neck tumors(HNC)

Head and neck cancers (HNC) consist of biologically similar malignant tumors located in the head and neck region, affecting areas like the oral cavity, nasal cavity, pharynx, and larynx. Over 90% of HNCs are squamous cell carcinomas, with the remaining cases mostly comprising adenocarcinomas, sarcomas, and lymphomas. In head and neck cancer (HNC), Gal-1 is extensively expressed and released into the surrounding environment. Patients exhibiting high Gal-1 expression in tumors or surrounding stroma show poorer responses and prognoses to immune checkpoint inhibitor therapy compared to those with lower Gal-1 levels [248]. Tumor-secreted Gal-1 in HNC appears to induce reprogramming of tumor endothelium, upregulating cell surface PD-L1 and Gal-9, creating an immunosuppressive barrier that prevents T cell infiltration [320].

In nasal cavity cancer tissue samples, Gal-4 expression generally increases, while in eosinophilic chronic rhinosinusitis and nasal polyps, Gal-4 shows lower levels of positive expression. Gal-7 is an effective marker for head and neck squamous cell carcinoma (HNSCC). It is highly expressed in various tumors, including squamous cell carcinoma of the buccal mucosa and esophagus, as well as sinonasal inverted papilloma [321]. In tongue squamous cell carcinoma, elevated Gal-7 expression is significantly linked to various tumor histological grades. The differential expression of Gal-7 in tumors of varying dysplasia grades is also accompanied by changes in subcellular localization [22]. Gal-7 can stimulate the expression of MMP-9 and MMP-2, facilitating the progression and metastasis of oral squamous cell carcinoma (OSCC). Gal-9 exhibits anti-tumor effects in esophageal squamous cell carcinoma (ESCC) and esophageal adenocarcinoma (EAC) by inducing apoptosis. Limited studies suggest that exogenous Gal-9 treatment can promote apoptosis in ESCC cells and regulate the JNK-p38 pathway to inhibit proliferation. In EAC, Gal-9 also induces apoptosis but may face resistance due to increased IL-8 expression [322].

### Neurological system tumors

Glioblastoma multiforme (GBM) is a prevalent form of malignant brain tumor. Gal-1 is expressed in all GBM types and serves as a key immunosuppressive factor. Silencing Gal-1 can reduce vascular density, promote immune cell infiltration, decrease the number of immunosuppressive MDSCs, and improve the secretion of immune-stimulatory cytokines [265]. On the other hand, GBM cells deficient in Gal-3 exhibit enhanced motility.

Neuroblastoma (NB) is an uncommon tumor that arises from embryonic neural crest cells. In individuals with recurrent and aggressive neuroblastoma (NB), Gal-1 expression is upregulated. NB cells secrete soluble Gal-1, which can induce T cell apoptosis and inhibit the maturation of DCs. In Gal-1 knockout mice, T cell infiltration is impaired, angiogenesis is inhibited, and tumor cell migration is reduced [323]. Gal-7 negatively regulates NB by controlling the transition of NB cells from proliferation to differentiation. Gal-8 facilitates the proliferation of U87 glioblastoma cells in vitro. Secreted into the culture medium, it interacts with extracellular glycans, promoting directed migration of U87 cells and inhibiting apoptosis, thus contributing to the malignant progression of glioblastoma cells [324].

In gliomas, the interaction between Gal-9 expressed by tumors and Tim-3 on T cells plays a crucial role. Activation of the Tim-3-Gal-9 pathway induces exhaustion of anti-tumor immune cells, the formation of M2-type tumor-associated macrophages, and promotes immune evasion of gliomas [325]. Gal-9 plays a vital role in the malignant progression of GBM, where elevated Gal-9 levels are significantly associated with decreased patient survival. In the glioma tumor microenvironment, isocitrate dehydrogenase (IDH) acts as a critical regulator of the Tim-3-Gal-9 pathway. IDH mutations can reduce the activity of the Tim-3-Gal-9 immune checkpoint pathway [326].

### Gastrointestinal tumors

#### Colorectal cancer (CRC)

Gal-2 expression, restricted to the gastrointestinal tract, is reduced in human CRC. Genome-wide CRISPR screening identified Gal-2 as an oxidative stress response gene that inhibits colorectal tumor growth. Increased Gal-2 levels inhibit the growth of human CRC epithelial cells and diminish H_2_O_2_-induced STAT3 phosphorylation [327].

In CRC patients, particularly those with metastasis, serum Gal-3 levels are markedly elevated. While Gal-3 expression in CRC tissues does not strongly correlate with clinical pathological parameters, it shows a positive association with levels of serum IL-17 and IL-23, suggesting a link between Gal-3 and tumor-related inflammation [328]. Gal-3 enhances the migratory capacity of CRC cells by activating the K-Ras/Raf/ERK pathway [329]. Inhibiting Gal-3 reduces the expression of its interacting protein, heterogeneous nuclear ribonucleoprotein Q (hnRNP Q), slowing cancer cell proliferation [330]. Gal-4 has been widely investigated in colorectal cancer, with a focus on its presence in the bloodstream and expression within tumor tissues. In CRC tissues and cells, Gal-4 expression is markedly reduced compared to normal colon tissues, and its levels are inversely related to cancer progression [68, 331]. However, in CRC patients, serum and plasma Gal-4 levels are higher compared to those in healthy individuals [332]. Gal-7 exhibits tumor-suppressive effects in CRC, inhibiting the proliferation and angiogenesis of CRC cell lines both in vitro and in vivo [22].

In CRC tissues, Gal-9 expression is reduced compared to the adjacent normal tissues. Low Gal-9 levels are positively associated with poor histological grade and lymph node metastasis in CRC. Gal-9 enhances NK cell recruitment, bolstering the body's immune surveillance against tumors. High levels of Gal-9 enhance apoptosis and suppress the growth of CRC cells. Tumor-secreted Gal-9 can induce apoptosis in Tim-3 positive CD8 T cells, but reducing cell surface Gal-9 can decrease Tim-3-Gal-9 pathway-mediated tumor immune evasion [288].

#### Gastric cancer (GC)

Low expression of Gal-2 is significantly linked to lymph node metastasis (LNM) and advanced clinical staging in GC (*P *= 0.024). Gal-2 expression is significantly higher in LNM-negative GC cases (*P* < 0.0001) [333]. In GC patients, serum Gal-3 levels are markedly higher than in healthy individuals and are linked to lymphatic metastasis. However, in some tumor tissues, Gal-3 expression is reduced, making it an unreliable biomarker for GC [51]. Gal-3 enhances metastasis in GC cells by increasing protease-activated receptor-1 (PAR-1) and MMP-1 and upregulating fascin-1. Suppressing Gal-3 decreases anti-apoptotic molecule expression and raises pro-apoptotic factors, thereby increasing the sensitivity of gastric cancer cells to chemotherapy.

Clinical evaluation of GC samples shows that Gal-4 expression is higher in gastric adenomas compared to healthy controls. Loss of Gal-4 is associated with lymphatic infiltration, N stage, and M stage in GC [334]. Studies on scirrhous GC cell lines suggest that Gal-4 expression may be related to its pro-metastatic effects [335]. Gal-4 is specifically expressed in the cytoplasm of poorly differentiated GC cells, promoting peritoneal dissemination of malignant cells [128]. In GC tissues, Gal-7 expression is decreased and significantly correlates with TNM staging and improved survival rates. There are methylation abnormalities in Gal-7 in GC cells, and demethylation treatment can increase Gal-7 expression. High levels of Gal-7 in GC cells inhibits cell proliferation, migration, and invasion [22, 307].

Gal-8 expression in GC is significantly linked with tumor size (*P* = 0.007), T stage (*P* = 0.001), N stage (*P* < 0.001), and TNM staging (*P* < 0.001). Cases with low Gal-8 expression have poorer OS (*P* < 0.001) and DFS (*P* < 0.001) [336]. A high level of Gal-9 expression is strongly linked to improved survival rates in GC. Patients with high Gal-9 expression have better tumor infiltration and lymph node metastasis profiles. Upregulating Gal-9 expression can inhibit invasion, migration, and EMT in GC cells. Recombinant human Gal-9 treatment induces apoptosis, modulates angiogenesis, and changes miRNA expression profiles, thereby inhibiting the proliferation of gastric cancer cells [288].

#### Pancreatic cancer

In pancreatic cancer tissues, Gal-3 expression levels are significantly elevated compared to normal tissues, adjacent non-tumor tissues, or benign pancreatic diseases. High Gal-3 levels are associated with poor differentiation and act as a pro-cancer factor in pancreatic cancer by binding and activating Ras, thereby promoting cancer progression. Additionally, Gal-3 regulates MUC1/EGFR-dependent pathways, stimulating pancreatic cancer cell growth [337]. In pancreatic cancer cell lines such as Capan 1, Gal-1 initiates its pro-anoikis activity signaling through the activation of caspase-8, while Gal-3 acts as an endogenous competitor of Gal-1 [338].

Pancreatic ductal adenocarcinoma (PDAC) is a highly aggressive cancer. In its stroma, Gal-1 is strongly expressed in activated pancreatic stellate cells (PSCs), which are a key component. Gal-1 triggers apoptosis in T cells and stimulates pancreatic stellate cells to secrete stromal cell-derived factor-1 (SDF-1), facilitating the migration and invasion of pancreatic cancer cells [339, 340]. Research utilizing tissue microarrays and immunohistochemical analysis has shown that Gal-4 expression is significantly associated with tumor size, differentiation status, postoperative recurrence, and survival rates. Studies have also reported on Gal-4 expression across different pancreatic cancer subtypes and cell lines. In the samples examined, PDAC and intraductal papillary mucinous carcinoma (IPMC) showed increased Gal-4 expression, while mucinous cystadenocarcinoma (MCAC) exhibited decreased Gal-4 expression. However, the limited sample size necessitates broader studies to confirm the relationship between Gal-4 expression and pancreatic cancer subtype classification [341]. In PDAC cell lines, Gal-4 expression appears to negatively correlate with cell migration properties. Reduced Gal-4 expression upregulates key Wnt/β-catenin pathway target genes, survivin and cyclinD1, leading to increased migration [21]. Recent research has emphasized the role of Gal-4 in modulating tumor immunity in PDAC. In patients with PDAC, elevated circulating Gal-4 levels are noted. Its presence in the tumor's extracellular matrix leads to T cell apoptosis by binding to N-glycosylated sites on CD3ε/δ, aiding in immune evasion [184].

Endogenous Gal-9 in pancreatic cancer has a pro-tumor effect, with high expression levels in both pancreatic cancer tissues and patient immune cells. Lower serum levels of Gal-9 are linked to extended survival durations [342]. Gal-9 encourages macrophages to differentiate into the M2 phenotype, decreases cytokine secretion such as TNF-α and IFN-γ, and diminishes anti-tumor immune responses. The interaction between Gal-9 and its receptor Dectin-1 also induces immune tolerance. Conversely, exogenous recombinant Gal-9 displays anti-tumor effects, promoting apoptosis in pancreatic cancer cells through the release of cytochrome c [343]. Gal-12 is significantly expressed in pancreatic cancer stromal cells and may participate in the dynamic crosstalk between cancer and stromal cells via a paracrine mechanism [344]. In primary leukemic cells from newly diagnosed AML patients, the promoter region of Gal-12 is highly methylated. Demethylation of these sites is required to induce Gal-12 expression. Low methylation of the Gal-12 promoter and its relative overexpression are associated with improved overall survival in AML patients [345].

#### Hepatocellular carcinoma

Hepatocellular carcinoma (HCC) is a highly heterogeneous malignant tumor. In HCC tissues, Gal-3 expression is significantly elevated, promoting HCC progression. Silencing Gal-3 in liver cancer cells inhibits their migration, RhoA-GTPase activity, and myosin light chain 2 phosphorylation. Downregulation of Gal-3 also reduces HCC cell proliferation and induces apoptosis. Gal-3 expression stimulates angiogenesis and can be used for prognostic evaluation, though it is less effective for diagnosing HCC patients [63]. Extensive studies have focused on the serum levels and tissue expression of Gal-4 in HCC. In reports focusing on the Chinese population, HCC patients affected by hepatitis B virus (HBV) show significantly elevated serum Gal-4 levels, with Gal-4 expression positively correlating with HBV viral load [207]. In HCC patients without HBV infection, Gal-4 levels do not show significant changes. In HCC cell lines such as Huh7, MHCC97L, and HCCLM3, Gal-4 expression is upregulated. The expression level of Gal-4 in HCC tissues is elevated compared to nearby non-cancerous tissues. The role of Gal-9 in HCC is dual-faceted. Gal-9, while impairing liver tumor immunity on one hand, also possesses anti-metastatic properties on the other. In HBV-associated HCC, Gal-9 positive Kupffer cells (KC) co-localize with Tim-3 positive T cells. Elevated Tim-3 expression on CD4 T cells correlates with reduced survival in HCC. Gal-9 also affects anti-tumor effector NK cells in the liver by downregulating multiple immune activation genes in NK cells. Contrary to its role in promoting tumor immune evasion, Gal-9's cytoplasmic presence stabilizes cell–cell adhesion, inhibiting HCC cell invasion and metastasis. Furthermore, Gal-9 induces apoptosis in HCC cells without relying on Tim-3 [193]. It is hypothesized that Gal-9 may be upregulated in the early stages of HCC to help establish an immune-tolerant microenvironment and later lost as the tumor progresses [346]. Abnormal Gal-14 expression is strongly linked to decreased overall survival in liver cancer patients. Suppressing Gal-14 expression can impede tumor development. Gal-14 promotes the expression of heparan sulfate proteoglycans (HSPGs) on the surface of HCC cells, increasing their responsiveness to growth factors and enhancing proliferation [166].

### Prostatic cancer

Gal-3 has been extensively studied in prostate cancer. Normal prostate epithelial tissue shows moderate Gal-3 immunostaining, which becomes more intense in prostatic intraepithelial neoplasia (PIN) but with a lower percentage of positive cells. In adenocarcinoma, Gal-3 expression is significantly reduced. Gal-3 is present in prostate cancer stem cells (CSCs) and lymph node metastases, potentially supporting tumor growth and metastatic spread through intracellular and extracellular mechanisms. In prostate cancer, Gal-3 exhibits immunosuppressive effects and promotes early metastasis. It contributes to the immune suppression mediated by prostate CSCs, enhancing their tumorigenic and metastatic capabilities. Intracellular expression of Gal-3 in prostate CSCs also increases the cells' resistance to apoptosis [317]. The levels of Gal-3 expression in malignant prostate epithelial cells is regulated by promoter methylation. Gal-3 inhibits mitochondria-mediated apoptosis by interacting with Bad, counteracting drug-induced cell death. Overexpression of Gal-3 inhibits calpain activation, reducing its pro-apoptotic function.

In prostate cancer, elevated Gal-4 expression is associated with cancer cell metastasis. Gal-4 interacts with the galactosyltransferase C1GALT1, affecting receptor tyrosine kinase (RTK) activation and influencing cell invasion and metastatic potential [347]. Downregulation of Gal-7 in prostate cells can inhibit cancer cell motility and reduce invasive behavior. The regulatory effect of Gal-7 on prostate cancer cell invasion depends on its CRD structure. Elevating Gal-7 levels can boost the sensitivity of prostate cancer cells to chemotherapy drugs [190]. Gal-8 is expressed in prostate cancer but not in normal prostate tissue and is implicated in the metastatic evolution of the disease. Gal-8 significantly impacts tumor cell anoikis resistance and homotypic aggregation by controlling cytoskeletal rearrangement and E-cadherin expression, thereby promoting the survival and metastasis of circulating tumor cells [214].

### Thyroid cancer

Thyroid cancer is both a frequent endocrine malignancy and a typical head and neck tumor. Papillary thyroid carcinoma (PTC) represents 70% of these cases. In PTC tumors and cell lines, Gal-2 expression is low. Elevated Gal-2 levels increase the sensitivity of PTC cells to apoptosis and inhibits PTC progression by activating the PI3K/Akt pathway [348]. Clinically, distinguishing between benign and malignant thyroid lesions is challenging. Gal-3 is a potential marker for differentiating PTC patients from non-PTC patients. Gal-3-positive PTC patients are more likely to experience lymph node metastasis [61]. Combining Gal-3 with other biomarkers like Hector Battifora mesothelial-1 (HBME-1) provides excellent sensitivity and specificity for diagnosing malignant thyroid lesions [349]. However, Several studies indicate that Gal-3 expression is not markedly linked to extrathyroidal extension, lymph node metastasis, overall metastasis, completeness of resection, invasiveness, or size classification [62].

Gal-7 shows differential expression across various types of thyroid tumors. In PTC, Gal-7 expression is upregulated, while it is downregulated in adenocarcinomas. This differential expression may help distinguish between benign and malignant thyroid cancers.

### Bladder cancer

Most bladder cancers are urothelial carcinomas, with a smaller proportion being squamous cell carcinomas. In highly differentiated squamous cell carcinomas, Gal-7 expression is elevated, while it is reduced in urothelial carcinomas. In urothelial carcinoma cells, Gal-7 can enhance sensitivity to the chemotherapy drug cisplatin (CDDP), regardless of the p53 mutation status. Gal-7 promotes the accumulation of ROS and the activation of JNK and Bax pathways [350]. A small study reported that Gal-3 expression is associated with the progression of urothelial carcinoma. Additionally, it has been suggested that the heterogeneity of Gal-3 within tumors complicates its use as a prognostic biomarker. Further investigation is needed to evaluate the potential assessment of galectins in serum and urine [351, 352].

## Potential therapeutic applications targeting galectins

**Fig. 8:**
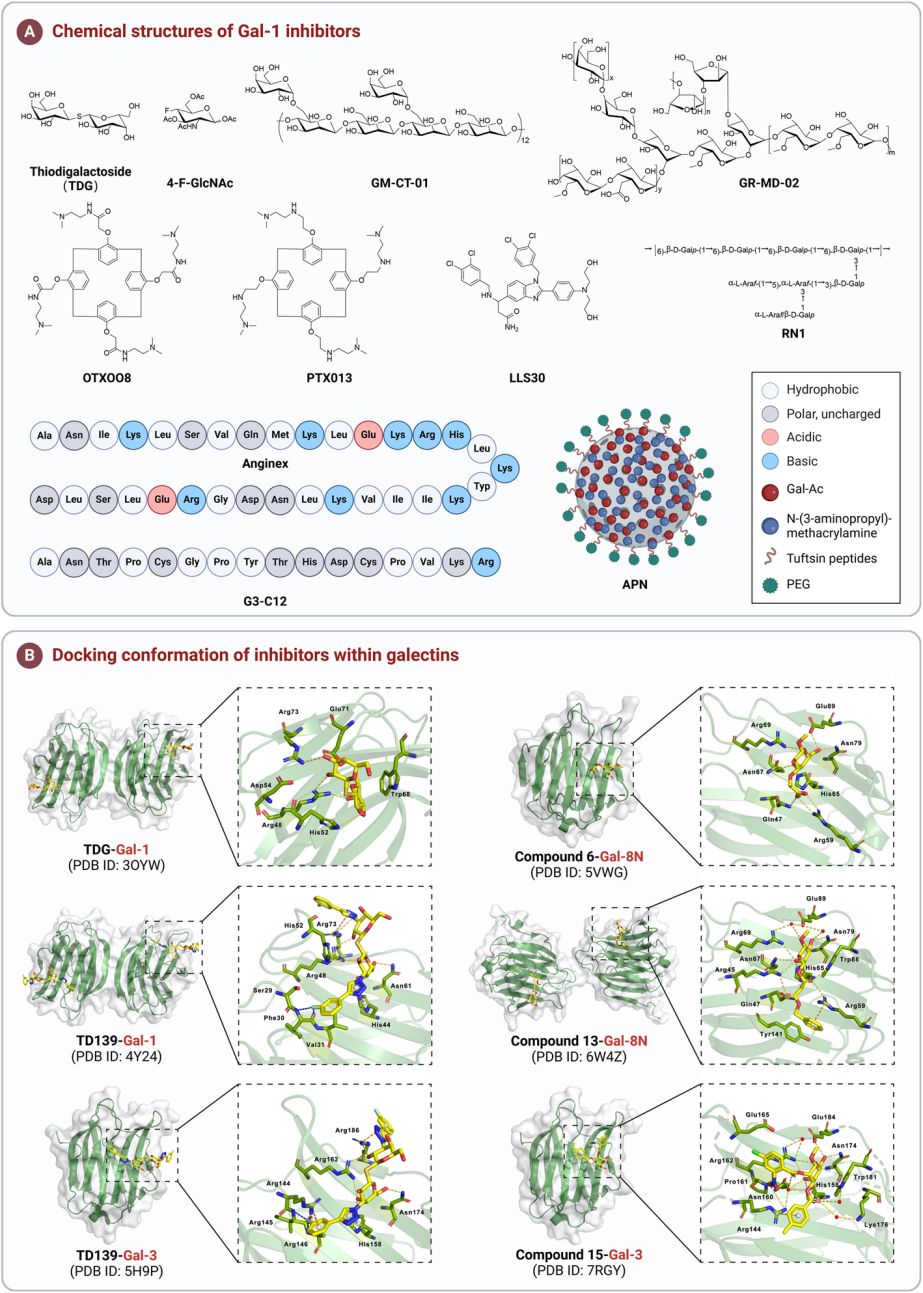
Therapeutic agents targeting Galectin. **a** Chemical structures of Gal-1 inhibitors. **b** Crystals of carbohydrate-recognition domains (CRDs) of human Galectins binding the small molecule inhibitors

Galectins seem to influence tumor progression at multiple levels, including angiogenesis, metastasis, and immune evasion. Various strategies have been employed to target galectins and disrupt their primary functions in tumor progression (Fig. 8 and Table 2) [159].

**Table 2:** Therapeutic agents targeting Galectin

Agents	Target (s)	Materials	Mechanisms	Models/Trials	Refs
Thiodigalactoside	Gal-1	Disaccharides	Competitively inhibit galectin-1 binding	Melanoma, breast cancer	[353, 354]
TD139 (GB0139)	Gal-1/3	Derivatives of TDG	Competitively inhibit galectin-1 binding	Idiopathic Pulmonary Fibrosis (Phase Ib/IIa)	NCT02257177 [355, 356]
4-F-GlcNAc	Gal-1	Glycan	Dampen the biosynthesis of LacNAcs	Melanoma, lymphoma	[6]
GM-CT-01 (Davanat)	Gal-1/3	Galactomannan	Bind to galectin-1 at a site opposite CRD, fuel IFN-γ secretion by TIL	Metastatic colorectal cancer (Phase I/II), Melanoma (Phase I/II)	NCT00054977, NCT00110721 and NCT01723813 [357, 358]
GR-MD-02 (Belapectin)	Gal-1/3	Polysaccharide	Remain obscure	NASH, Non-alcoholic fatty liver disease	NCT02421094, NCT02462967 and NCT01899859
				Head and neck cancer, melanoma combined with pembrolizumab (Phase I)	NCT02575404 [359]
MCP (GCS-100)	Gal-1/3	Polysaccharide	A galectin-3 inhibitor	Prostate cancers, liver metastasis of colon cancer	[360, 361]
SLA	Gal-1/3	Lactulose amines	Bind to galectin-1 and galectin-3	Lung carcinoma, melanoma	[362]
RN1	Gal-3	Polysaccharide	Bind to galectin-3 and suppresses its expression	Pancreatic ductal adenocarcinoma	[363]
Methyl 3-O-[1-carboxyethyl]-β-d-galactopyranoside (compound 6)	Gal-8N	Monosaccharide-based scaffold	Bind to galectin-8N and inhibit galectin-8N	–	[364]
Methyl-β-d-galactomalonyl Phenyl Esters	Gal-8N	Monosaccharide-based compounds	Bind to galectin-8N and inhibit galectin-8N	Breast cancer cell line	[365]
N-Arylsulfonyl-Indole-2-Carboxamide Derivatives	Gal-3/8C	Small molecule	Dual inhibit galectin-3 and galectin-8 C-terminal domain	Lung fibroblast cells	[366]
methyl 2-O-(2-nitro-4-chloro-benzoyl)−3-O-toluoyl-β-d-talopyranoside	Gal-3	Monosaccharide inhibitor	Inhibit galectin-3	B-cell precursor acute lymphoblastic leukemia	[367]
Anginex	Gal-1/2/7/8/9	β-peptide	Alter the equilibrium of galectin-ligand binding	Murine ovarian carcinoma model	[368]
TFD100	Gal-3	Glycopeptide	Bind galectin-3 with picomolar affinity and blocks its functions	Prostate cancer	[369]
G3-C12	Gal-3	Peptide	Specifically binds to the carbohydrate-recognition domain of galectin-3	Prostate cancer	[370]
Galectin-3C	Gal-3	Truncated, dominant negative form of galectin-3	Block endogenous galectin-3	Ovarian cancer	[371]
OTX008	Gal-1	Calixarene compound	Allosteric inhibitor of galectin-ligand binding	Human advanced solid tumors (Phase I)	NCT01724320 [372]
LLS30	Gal-1	Small molecule	Allosteric inhibitor of galectin-ligand binding	Prostate cancer, hepatocellular carcinoma	[373]
PTX013	Gal-1	Small molecule (Polycationic calixarene)	Allosteric inhibitor of galectin-ligand binding	Human cancer cell lines and drug resistant cancer cells	[374]
AP-74 M-545	Gal-1	Single-stranded DNA aptamer	Impair galectin-ligand binding	Murine lung cancer model	[375]
8F4F8G7	Gal-1	Monoclonal antibody	Eliminate galectin-1 in tumor tissue	Kaposi’s sarcoma, prostate cancer	[376]
Gal-1-mAb3	Gal-1	Monoclonal antibody	Antibody with higher affinity and selectivity	-	[377]
APN	Gal-1	Antibody-like polymeric nanoparticle	Eliminate galectin-1 in tumor tissue	-	[378]
TRX–mGal1	Gal-1	Murine galectin-1 vaccine	Induce generation of endogenous antibody	Melanoma	[379]
Minigene DNA vaccine	Gal-1Ta	DNA plasmid	Encode peptide fragment of galectin-1	Neuroblastoma	[380]
Intranasal siRNA	Gal-1	siRNA-loaded chitosan nanoparticles	Inhibit galectin-1 expression	Glioblastoma multiforme	[381]

Since galectins recognize galactose or galactose-containing oligosaccharides, most drug discoveries have focused on synthesizing and modifying galactosides, lactoses, and their analogs, such as monovalent carbohydrates like lactose and thiodigalactoside (TDG). TDG is one of the most successful carbohydrate-based galectin inhibitors, exhibiting higher affinity for Gal-1 than natural ligands. By counteracting Gal-1's immunosuppressive effects, it enhances the vaccine-stimulated anti-tumor immune response in vivo. TDG also promotes the infiltration of anti-tumor immune cells and inhibits tumor angiogenesis [156]. Additionally, TDG has been shown to inhibit the angiogenic activity of Gal-8, suggesting it may serve as a broad-spectrum anti-angiogenic compound [1]. TD139 (GB0139), a derivative of TDG with high specificity for Gal-1 and Gal-3, targets the glycan-binding pockets of galectins. It has shown effectiveness in phase Ib/IIa clinical trials for treating idiopathic pulmonary fibrosis (ClinicalTrials.gov: NCT02257177) [355, 356]. Further modifications of TDG (GB0139) have led to the development of compounds with higher oral bioavailability, such as GB1211, GB1490, and GB1908 [382]. Overacetylated 4-fluoro-glucosamine (4-F-GlcNAc) can restrict LacNAc biosynthesis, reducing Gal-1 binding to LacNAc on T cell membrane proteins, thus inhibiting melanoma and lymphoma development [383]. Additionally, glycans can serve as targeting moieties to deliver therapeutic drugs to galectin-rich cancer cells [159]. Lactosyl-L-leucine also inhibits Gal-3, displaying anti-metastatic and pro-apoptotic effects [384, 385]. Due to the reliance of intercellular recognition and signal transduction on multivalent receptor-ligand complexes, there has been significant interest in developing multivalent carbohydrate-based inhibitors. Most multivalent inhibitors are designed to target Gal-3, such as the polysaccharides GM-CT-01 (Davanat) and GR-MD-02 (Belapectin) developed by Galectin Therapeutics, which have been patented as cancer treatments [386–389]. GM-CT-01 and GR-MD-02 interact with both Gal-1 and Gal-3. In phases I and II clinical trials, the combination of GM-CT-01 and 5-fluorouracil enhanced cancer treatment outcomes (ClinicalTrials.gov: NCT00054977, NCT00110721, and NCT01723813). In a recent phase I study, the combination of GR-MD-02 and the anti-PD-1 drug pembrolizumab demonstrated encouraging clinical responses in head and neck squamous cell carcinoma as well as metastatic melanoma (ClinicalTrials.gov: NCT02575404). GR-MD-02 is a complex polysaccharide similar in composition to modified citrus pectin (MCP, GCS-100), a galactose-rich polysaccharide that inhibits Gal-3 binding to endothelial cells, reducing prostate cancer cell viability and enhancing their sensitivity to radiotherapy. Synthetic lactulose amines (SLA) derivatives also bind to Gal-1 and Gal-3 with varying affinities, inhibiting endothelial cell tube formation [362]. RN1, another polysaccharide that binds to and inhibits Gal-3 expression, significantly suppresses the growth of PDAC cells both in vitro and in vivo [363]. Gal-3 can interact with the short β −1,4-galactan side chains in the pectin RG-I-4 derived from ginseng. RG-I-4 can inhibit Gal-3 function, significantly promote T cell proliferation and IL-2 expression, and inhibit tumor growth [390].

In addition to synthetic or naturally occurring carbohydrates, protein- and peptide-based inhibitors can also block the biological activity of galectins. Anginex, a synthetic β-peptide, binds to the β-sheet motif of Gal-1, inhibiting endothelial cells and impairing tumor microvasculature formation [368]. Anginex also has affinity for Gal-2, −7, −8, and −9. Although the precise mechanism of anginex is unclear, it likely alters the carbohydrate affinity of galectins through protein–protein interactions. Evidence also suggests that anginex can block downstream signaling by inhibiting the membrane translocation of activated H-Ras.

TFD100, a glycopeptide with picomolar affinity for Gal-3, can block Gal-3-mediated angiogenesis and metastasis in prostate cancer cells [369]. A Gal-3-targeting N-(2-hydroxypropyl) methacrylamide copolymer (G3-C12)−5-fluorouracil conjugate significantly enhances the antitumor activity of 5-fluorouracil [370]. Gal-3C, a truncated negative form of Gal-3, can endogenously block Gal-3, significantly reducing the proliferation and invasion capabilities of ovarian cancer cells [371]. Moreover, a novel small-molecule allosteric inhibitor of Gal-1, OTX008 (0018), can disrupt Gal-1 interactions with cell surface carbohydrates [372]. This small non-peptide molecule has demonstrated effectiveness in normalizing tumor vasculature and decreasing tumor proliferation and invasion in both cell and animal models. PTX013, a derivative of OTX008, inhibits the growth of several human tumor cells and resistant cell lines [374]. LLS30, another Gal-1 allosteric inhibitor, effectively inhibits the invasive properties of prostate cancer and cancer stem cells [373]. Currently, no FDA-approved drugs target Gal-1, indicating that more research is needed in galectin-targeted therapies.

The single-stranded DNA aptamer AP-74 M-545 specifically antagonizes Gal-1. In a mouse lung cancer model, AP-74 M-545 blocked the interaction between CD45 and Gal-1, preventing the apoptosis of tumor-infiltrating T cells [375]. Monoclonal antibodies inhibit tumor angiogenesis and regression by blocking target molecules. This includes Gal-1-mAb3 and APN (antibody-like polymer nanoparticle), which neutralize Gal-1's functions. The murine galectin-1 vaccine (TRX-mGal1) induces anti-Gal-1 antibodies, reducing tumor growth. Mini-gene DNA vaccines generate antibodies against galectins, and siRNA-based silencing disrupts galectin expression. For other types of galectin inhibitors, see Table 2. Other members of the galectin family, such as Gal-7, have also been targeted by inhibitors, including several small-molecule carbohydrate and non-carbohydrate inhibitors. These inhibitors and their derivatives show higher affinity for Gal-7 than other galectins, though their potential clinical applications need further evaluation in cell and animal models [391].

Compound polysaccharides developed to target galectins have achieved some success, with natural polysaccharides showing good tolerance and safety in clinical use for various diseases, including cancer. However, attributing clinical efficacy to the inhibition of a single target is not rigorous due to the broad impact of galectins on multiple diseases. These inhibitors are promising for diseases where galectins play a primary role, but further evaluation is needed for treating multi-target related diseases. The development of galectin-targeted therapies faces challenges, such as protein specificity and the unfavorable pharmacokinetics of protein-based therapeutics.

## Conclusions and perspective

This review highlights the crucial roles of galectins in cancer biology, including tumor cell proliferation, transformation, metastasis, angiogenesis, and immune response within tumors. The diverse structural configurations and multivalent binding capabilities of Galectins enable them to regulate various signaling pathways and cellular functions with complexity and precision across different spatiotemporal levels, making them integral components of cancer progression. Our analysis indicates that Galectins are not only essential for understanding tumor biology but also hold promise as therapeutic targets.

The importance of Galectins in early cancer detection, personalized therapy, and predicting treatment responses cannot be overstated. By elucidating the mechanisms through which Galectins influence cancer development and progression, researchers can identify novel biomarkers and develop targeted therapies that improve treatment efficacy and patient outcomes. Insights gained from studying Galectin interactions and functions provide a robust foundation for advancing cancer treatment strategies. Considering the complexity of Galectin functions, future research should focus on overcoming current challenges in Galectin-targeted therapies, such as specificity and delivery mechanisms. Additionally, expanding our understanding of the roles of Galectins in different cancer types of cancer and their interactions with other cellular components will be crucial. Integrating Galectin research into clinical practice holds promising prospects for cancer diagnosis and treatment, ultimately leading to more effective and personalized patient care. In summary, the ongoing exploration of Galectin functions and their implications in cancer biology will continue to be a pivotal area of research. As we deepen our knowledge, the potential to translate these findings into clinical applications promises significant advancements in the fight against cancer.

## Data Availability

No datasets were generated or analysed during the current study.

## Abbreviations

AML: Acute Myeloid Leukemia
ATLL: Adult T-Cell Leukemia/Lymphoma
APL: Acute Promyelocytic Leukemia
Bcl-2: B-Cell Lymphoma-2
CCRT: Concurrent Chemoradiotherapy
C/EBPb: CCAAT/Enhancer-Binding Protein Beta
CD44: Cluster Of Differentiation 44
CD40: Cluster Of Differentiation 40
CLCs: Charcot-Leyden Crystals
CML: Chronic Myeloid Leukemia
CRC: Colorectal Cancer
CSCs: Cancer Stem Cells
CSF1: Colony-Stimulating Factor 1
CSF1R: CSF1 Receptor
CTCL: Cutaneous T-Cell Lymphoma
CTCs: Circulating Tumor Cells
DCs: Dendritic Cells
DFS: Disease-Free Survival
DLBCL: Diffuse Large B-Cell Lymphoma
ECM: Extracellular Matrix
EBV: Epstein-Barr Virus
EAC: Esophageal Adenocarcinoma
EMT: Epithelial-Mesenchymal Transition
ER: Estrogen Receptors
ESCC: Esophageal Squamous Cell Carcinoma
FAK: Focal Adhesion Kinase
FL: Low-Grade Follicular Lymphoma
Gal: Galectin
GlcNAc: N-Acetylglucosamine
Glut-2: Glucose Transporter 2
GTP: Guanosine-5'-Triphosphate
HBME-1: Hector Battifora Mesothelial-1
HA: Hyaluronic Acid
HCC: Hepatocellular Carcinoma
HMMR: Hyaluronic Acid-Mediated Motility Receptor
HNSCC: Head And Neck Squamous Cell Carcinoma
hnRNP Q: Heterogeneous Nuclear Ribonucleoprotein Q
HNC: Head And Neck Cancers
HSPGs: Heparan Sulfate Proteoglycans
HSF-1: Heat Shock Factor-1
HPV: Human Papillomavirus
HSIL: High-Grade Squamous Intraepithelial Lesions
IDH: Isocitrate Dehydrogenase
IPMC: Intraductal Papillary Mucinous Carcinoma
IRS: Immunoreactivity Score
IS: Immune Synapse
KC: Kupffer Cells
LAG3: Lymphocyte Activation Gene 3
LacNAc: N-Acetyllactosamine
LECs: Lymphatic Endothelial Cells
LGALS3BP: Lectin-Galactoside Binding Soluble 3-Binding Protein
LILRB4: Leukocyte Immunoglobulin-Like Receptor B4
LMP-1: Epstein-Barr Virus Latent Membrane Protein-1
LNM: Lymph Node Metastasis
LTA: Lymphotoxin-α
MDSCs: Myeloid-Derived Suppressor Cells
MCAC: Mucinous Cystadenocarcinoma
MCAM: Melanoma Cell Adhesion Molecule
MDCK: Madin-Darby Canine Kidney Epithelial Cells
MMP-1: Matrix Metalloproteinase-1
MMP-9: Matrix Metalloproteinase-9
MM: Metastatic Melanoma
MSCs: Mesenchymal Stromal Cells
MUC1: Mucin 1
NB: Neuroblastoma
NHL: Non-Hodgkin Lymphoma
NSCLC: Non-Small Cell Lung Cancer
OS: Overall Survival
OSCC: Oral Squamous Cell Carcinoma
PAR-1: Protease-Activated Receptor-1
PDAC: Pancreatic Ductal Adenocarcinoma
PDPN: Podoplanin
pSMAC: Peripheral Supramolecular Activation Cluster
PIN: Prostatic Intraepithelial Neoplasia
PI3K: Phosphatidylinositol 3-Kinase
PR: Progesterone Receptors
PSCs: Pancreatic Stellate Cells
PTC: Papillary Thyroid Carcinoma
PGE2: Prostaglandin E2
ROS: Reactive Oxygen Species
RS: Reed-Sternberg
SCC: Squamous Cell Carcinoma
SG: Stress Granule
SDF-1: Stromal Cell-Derived Factor-1
SLA: Synthetic Lactulose Amines
SCLC: Small Cell Lung Cancer
TCR: T Cell Receptor
TAMs: Tumor-Associated Macrophages
TDG: Thiodigalactoside
Th1: T Helper Type 1
TILs: Tumor-Infiltrating Lymphocytes
TICs: Tumor-Initiating Cells
TID1: Tumorous Imaginal Disc
Tim-3: T Cell Immunoglobulin Domain And Mucin Domain-3
TLR-4: Toll-Like Receptor 4
TNBC: Triple-Negative Breast Cancer
Tn: Thomsen-Nouveau
VISTA: V-Domain Ig-Containing Suppressor Of T Cell Activation
